# Interleukin13 Ameliorates Hepatocellular Steatosis via **IL13Rα1/JNK/FOSL2** Signaling Pathway

**DOI:** 10.1016/j.jcmgh.2026.101879

**Published:** 2026-09-03

**Authors:** Verena von Bülow, Dejan A. Felske, Sarah B. Riebeling, Fabian P. Schmidt, Frederik Stettler, Andreas Schmid, Thomas Karrasch, Klaus Eder, Erika Most, Gertrud Morlock, Annika Haase, Jens Bier, Heike M. Hermanns, Andreas Geier, Christopher Tuffs, Jörn Pons-Kühnemann, Martin Roderfeld, Elke Roeb

**Affiliations:** 1 Department of Gastroenterology, Justus Liebig University, Giessen, Germany; 2 Department of Internal Medicine III, Justus Liebig University, Giessen, Germany; 3 Institute of Animal Nutrition and Nutritional Physiology, Justus Liebig University, Giessen, Germany; 4 Institute of Nutritional Science, Food Science Department, and Interdisciplinary Research Center (iFZ), Justus Liebig University, Giessen, Germany; 5 Cardio-Pulmonary Institute, Justus Liebig University, Giessen, Germany; 6 Division of Hepatology, University Hospital Würzburg, Würzburg, Germany; 7 Clinic for General, Visceral, Thoracic and Transplant Surgery, University Hospital of Giessen, Giessen, Germany; 8 Medical Statistics, Institute of Medical Informatics, Justus Liebig University, Giessen, Germany

**Keywords:** FOSL2, IL13, Lipid Metabolism, MASH, MASLD, Obesity

## Abstract

**Background & Aims:**

Severe obesity is a major global health challenge and a key driver of hepatic steatosis through systemic insulin resistance and enhanced de novo lipogenesis. Excessive hepatic triglyceride accumulation represents a central metabolic consequence of this condition. This study aimed to identify immune-derived regulators of liver lipid metabolism and to define the mechanistic role of interleukin13 (IL13) in obesity-associated liver disease progression.

**Methods:**

IL13 levels and related signaling pathways were analyzed in plasma samples from patients with ≥class II obesity and liver biopsies from patients with hepatic steatosis and steatohepatitis. The effects of IL13 depletion on lipid metabolism were examined in mice, whereas its mechanistic role in fat metabolism was studied in primary hepatocytes.

**Results:**

In a cohort of severely obese patients, plasma IL13 levels rose after weight loss and were positively associated with fibroblast growth factor 19 (FGF19), fibroblast growth factor 21 (FGF21), and insulin-like growth factor-1 (IGF1). IL13-deficient mice showed worsened liver triglyceride accumulation, lower polyunsaturated fatty acids, and altered expression of peroxisome proliferator–activated receptor–dependent lipid genes. Primary murine hepatocytes revealed that IL13 decreases triglyceride content through interleukin13Rα1 (IL‑13Rα1) and a c-Jun N-terminal kinase (JNK)–dependent c-JUN–FOS-like antigen 2 (FOSL2) axis, which enhances β-oxidation and ketogenesis. FOSL2 was required for maximal peroxisome proliferator–activated receptor (PPAR) activity and was upregulated in livers of patients with hepatic steatosis and steatohepatitis.

**Conclusions:**

These data demonstrate that IL13 is a critical immunometabolic regulator of hepatic lipid homeostasis. Targeting the IL‑13-FOSL2-PPAR signaling pathway may offer a novel therapeutic approach to reduce hepatic steatosis.

## Graphical abstract

**Figure:**
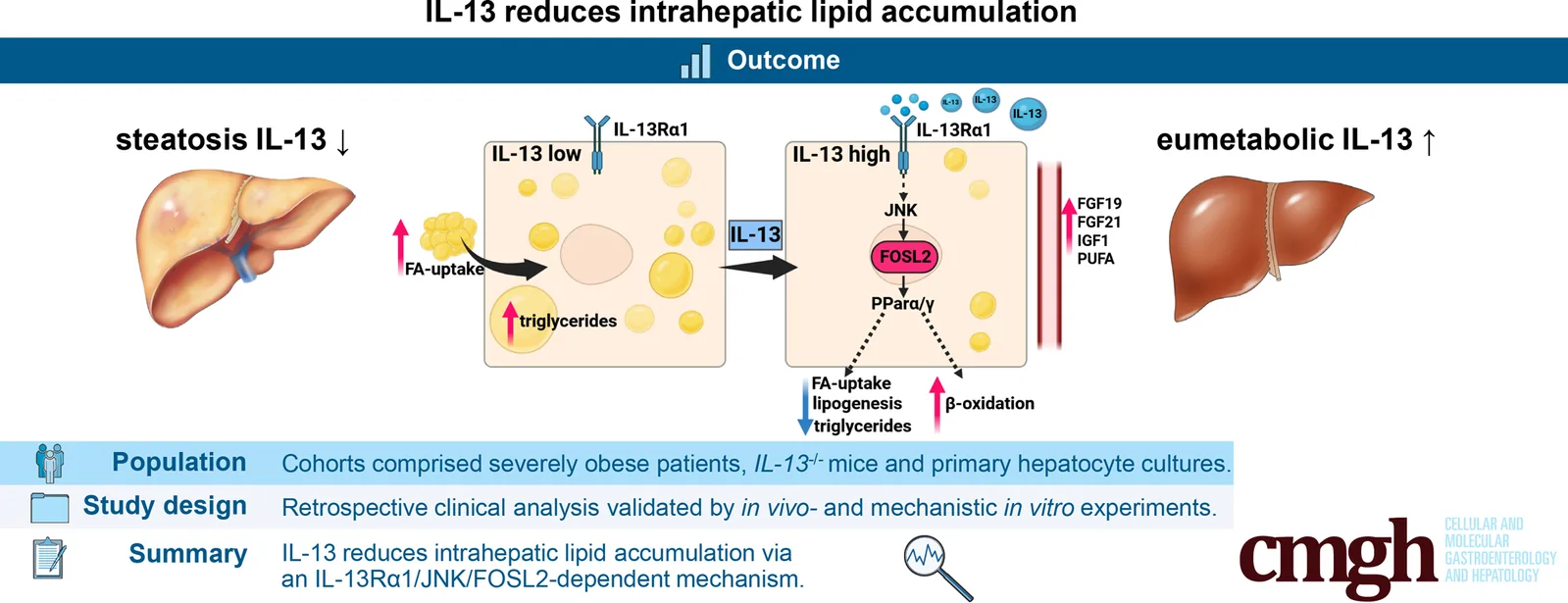

**Summary**

Cytokines are recognized as mediators linking metabolic and inflammatory pathways. IL13 reduces intrahepatic lipid accumulation via an IL13Rα1/JNK/FOSL2-dependent mechanism.

**What You Need to Know**

**Background**

Cytokines are recognized as mediators linking metabolic and inflammatory pathways. The role of IL13 and its mode of action in hepatic steatosis remain unclear.

**Impact**

IL13 reduces intrahepatic lipid accumulation via an IL13Rα1/JNK/FOSL2–dependent mechanism. IL13 enhances fatty acid β-oxidation, promotes ketogenesis, and modulates key metabolic regulators.

**Future Directions**

These findings highlight IL13 as a promising therapeutic target for the treatment of metabolic dysfunction–associated steatotic liver disease.

Steatotic liver disease has emerged as a major global health challenge, mirroring the rise in obesity and type 2 diabetes.^1^ The condition is defined by hepatic steatosis, characterized by triglyceride (TG) accumulation within hepatocytes.^2^ This process is primarily driven by enhanced hepatic de novo lipogenesis secondary to insulin resistance and by an increased influx of free fatty acids from adipose tissue.^3^ Steatosis ensues when lipid acquisition exceeds the liver’s capacity for β-oxidation and export.^3^ Persistent lipid overload may provoke lipotoxicity, inflammation, and fibrotic remodeling, culminating in steatohepatitis.^4^ Chronic low-grade inflammation, mediated in part by cytokines, is now recognized as central to disease progression.^5^

Among these mediators, interleukin13 (IL13) has attracted growing interest. Traditionally associated with allergic inflammation and tissue repair, IL13 exerts pleiotropic effects on epithelial, immune, and mesenchymal cells, promoting extracellular matrix deposition and, in pathologic contexts, fibrosis.^6^ Signaling via interleukin13 receptor α1 (IL13Rα1) in concert with the IL4 receptor α chain activates signal transducer and activator of transcription 6,^7^ whereas IL13Rα2 enhances profibrotic transforming growth factor-β1 (TGF-β1) pathways. Intriguingly, IL13 also modulates glucose metabolism through IL13Rα1 and signal transducer and activator of transcription 3 (STAT3).^8^

Accumulating evidence implicates the important role of transcription factor activator protein-1 (AP-1), particularly members of the FOS family, in the pathogenesis of steatotic liver disease. Enhanced expression of FOS-like antigen 1 (FOSL1) and FOS-like antigen 2 (FOSL2; FRA) has been shown to confer protection against diet-induced steatotic liver injury.^9^ This effect is mediated through suppression of peroxisome proliferator-activated receptor γ (PPARγ) transcription via inhibitory heterodimers formed between c-JUN and either FOSL1 or FOSL2.^9^ Moreover, central pathogenic processes, including insulin resistance, endoplasmic reticulum stress, and autophagy, are coordinated by the upstream kinases c-Jun N-terminal kinase (JNK)1 and JNK2, which regulate c-JUN activity.^10^

This pathway attenuates lipid uptake and limits triglyceride synthesis in hepatocytes, promotes β-oxidation in vitro*,* and is associated with increased ketogenesis in patients with severe obesity, whereas FOSL2 induction is observed in liver tissue from patients with hepatic fat accumulation.

## Results

### Plasma IL13 Was Associated With Body Mass Index, FGF19, FGF21, and IGF1 in Obese Patients

IL13 plasma concentrations were measured in patients with class II and III obesity at baseline and after a 3-month follow-up. Baseline characteristics are shown in Tables 1 and 2.

**Table 1:** Baseline Characteristics of the Study Population Subdivided According to Sex and IL13 Levels

Variable	Female – low IL13	Female – high IL13	Male – low IL13	Male – high IL13
IL13, *pg/mL*	35.6 [0.0–89.5] (40)	733 [109–37,010] (148)	70.4 [39.0–94.4] (8)	575 [126–22,207] (56)
Age, *y*	38.0 [22.0–60.0] (40)	37.0 [19.0–69.0] (148)	41.0 [25.0–58.0] (8)	42.5 [19.0–73.0] (56)
Perimenopausal (age ≥45 and <52 y)	22.5 (9)	18.9 (28)		
Postmenopausal (age ≥52 y)	17.5 (7)	12.2 (18)		
BMI, *kg/m*^*2*^	48.7 [38.4–60.7] (40)	49.9 [35.6–83.7] (148)	57.2 [43.8–61.1] (8)	49.1 [36.1–81.5] (56)
Body weight, *kg*	130 [90.9–163] (40)	134 [90.2–239] (148)	174 [142–211] (8)	159 [116–264] (56)
Body fat	52.1 [39.5–62.1] (33)	53.8 [37.6–65.7] (135)	40.4 [38.9–46.9] (6)	41.1 [29.3–52.0] (49)
Hyperlipidemia	42.5 (17)	35.1 (52)	50.0 (4)	55.4 (31)
Gastric bypass	60.0 (24)	52.7 (78)	25.0 (2)	32.1 (18)
Gastric sleeve	5.0 (2)	9.5 (14)	50.0 (4)	12.5 (7)
Low calorie diet	35.0 (14)	37.8 (56)	25.0 (2)	55.4 (31)
Hemoglobin A1c	5.70 [4.40–8.30] (36)	5.60 [4.70–9.90] (138)	5.50 [4.70–6.70] (7)	5.85 [4.7–9.0] (52)
Type 2 diabetes	17.5 (7)	16.9 (25)	25.0 (2)	30.4 (17)
TG, *mg/dL*	125 [58.0–513] (38)	123 [47.0–429] (141)	177 [70.0–239] (7)	145 [48.0–4326] (52)
Cholesterol, *mg/dL*	176 [108–288] (38)	186 [115–268] (141)	140 [115–186] (7)	181 [104–290] (52)
HDL, *mg/dL*	48.0 [26.0–87.0] (38)	46.0 [17.0–124] (141)	33.0 [29.0–43.0] (7)	39.0 [25.0–63.0] (52)
LDL, *mg/dL*	125 [42.0–209] (38)	125 [11.0–212] (141)	95.0 [53.0–131] (7)	128 [53.0–233] (52)
Glucose, *mg/dL*	97.0 [72.0–215] (39)	92.0 [73.0–298] (146)	100 [84.0–155] (7)	99.0 [77.0–291] (55)
Insulin, *mU/L*	24.9 [13.4–201] (37)	21.9 [6.00–152] (124)	43.4 [24.8–88.9] (6)	31.4 [4.40–500] (50)
HOMA-IR	5.9 [2.85–70.1] (37)	5.03 [1.23–74.0] (124)	8.85 [5.93–22.0] (5)	7.84 [1.20–186] (50)
<2.5 (no IR)	–	8.1 (10)	–	86.0 (43)
2.5–4.0 (moderate)	24.3 (9)	29.8 (37)	–	8.0 (4)
>4.0 (severe IR)	75.7 (28)	62.1 (77)	100 (5)	6.0 (3)
CRP, *mg/dL*	10.8 [0.62–37.8] (40)	10.4 [0.50–111] (147)	10.4 [2.39–31.7] (8)	8.95 [1.48–267] (55)
FGF19, *pg/mL*	43.9 [3.25–196] (23)	82.3 [9.49–1307] (117)	35.5 [4.42–155] (6)	74.5 [5.27–900] (43)
FGF21, *pg/mL*	203 [23.8–681] (22)	258 [17.3–5480] (118)	132 [0.0–788] (7)	313 [35.9–3252] (44)
IGF1, *pg/mL*	512 [294–965] (14)	553 [77.7–2855] (86)	507 [222–2623] (6)	564 [73.0–2417] (37)
Hormonal contraception	30 (12)	21.6	–	–
Smoking	27.5 (11)	28.6 (42)	62.5 (5)	28.6 (16)
Hypertension	61.5 (24)	45.2 (66)	75.0 (6)	72.2 (39)
Childhood obesity	72.5 (29)	73.5 (108)	62.5 (5)	67.9 (38)

NOTE. Data are presented as median [range] (n) or percentage (n).

Number of observations (n) varies due to missing data (exact n in parentheses).

Fourteen patients with IL13 <11.7 pg/mL were excluded from calculation.

Perimenopausal and menopausal ages from.^11^

BMI, body mass index; CRP, C-reactive protein; FGF, fibroblast growth factor; HDL, high-density lipoprotein; HOMA-IR, Homeostatic Model Assessment for Insulin Resistance; IGF1, insulin-like growth factor-1; IL13, interleukin13; LDL, low-density lipoprotein; TG, triglyceride.

**Table 2:** Baseline Liver Parameters of the Study Population Subdivided According to Sex and IL13 Levels

Variable	Female – low IL13	Female – high IL13	Male – low IL13	Male – high IL13
ALT, *U/L*	15.8 [4.00–36.8] (30)	14.2 [4.20–46.1] (138)	12.7 [5.90–31.6] (4)	18.7 [5.90–54.3] (50)
NFS	−1.36 [−3.03 to 0.42] (12)	−1.42 [−3.65 to 2.71] (52)	−0.45 [−1.66 to 0.76] (2)	−1.14 [−2.98 to 2.02] (28)
<−1.455 (no fibrosis)	41.7 (5)	50 (26)	50 (1)	32.1 (9)
−1.455 to 0.675 (intermediate)	58.3 (7)	42.3 (22)	–	53.6 (15)
>0.675 (advanced fibrosis)	–	7.7 (4)	50 (1)	14.3 (4)
ALBI	−2.25 [−2.98 to 1.52] (18)	−2.28 [−2.75 to 1.50] (54)	−2.24 [−2.25 to 2.22] (2)	−2.28 [−2.91 to 1.48] (28)
Grade 1 (≤−2.60)	22.2 (4)	14.8 (8)	–	25 (7)
Grade 2 (−2.60 to −1.39)	77.8 (14)	85.2 (46)	100 (2)	75 (21)
Grade 3 (>−1.39)	–	–	–	–
BARD	3.00 [1–4] (20)	1.00 [1–4] (56)	1.5 [1–2] (2)	1.00 [1–4] (28)
0–1 (low risk)	20 (4)	53.6 (30)	50 (1)	57.1 (16)
2–4 (high risk)	80 (16)	46.4 (26)	50 (1)	42.9 (12)
FIB-4	0.70 [0.42–1.27] (16)	0.58 [0.16–1.75] (52)	0.48 [0.41–0.55] (2)	0.68 [0.31–3.00] (26)
<1.3 (low risk)	100 (16)	96.2 (50)	100 (2)	80.8 (21)
1.3–2.67 (intermediate risk)	–	3.8 (2)	–	15.4 (4)
>2.67 (high risk)	–	–	–	3.8 (1)

NOTE. Data are presented as median [range] (n) or percentage (n).

Number of observations (n) varies due to missing data (exact n in parentheses).

ALBI, Albumin-Bilirubin; ALT, alanine aminotransferase; AST, aspartate aminotransferase; BARD, BMI-AST/ALT-Ratio-Diabetes; BMI, body mass index; FIB-4, Fibrosis-4 index; IL13, interleukin13; NFS, NAFLD Fibrosis Score.

The female cohort comprised mainly bariatric surgery patients, with the remainder following a low-calorie diet (Methods). IL13 levels increased after 3 months of intervention (Figure 1*A*).

**Figure 1:**
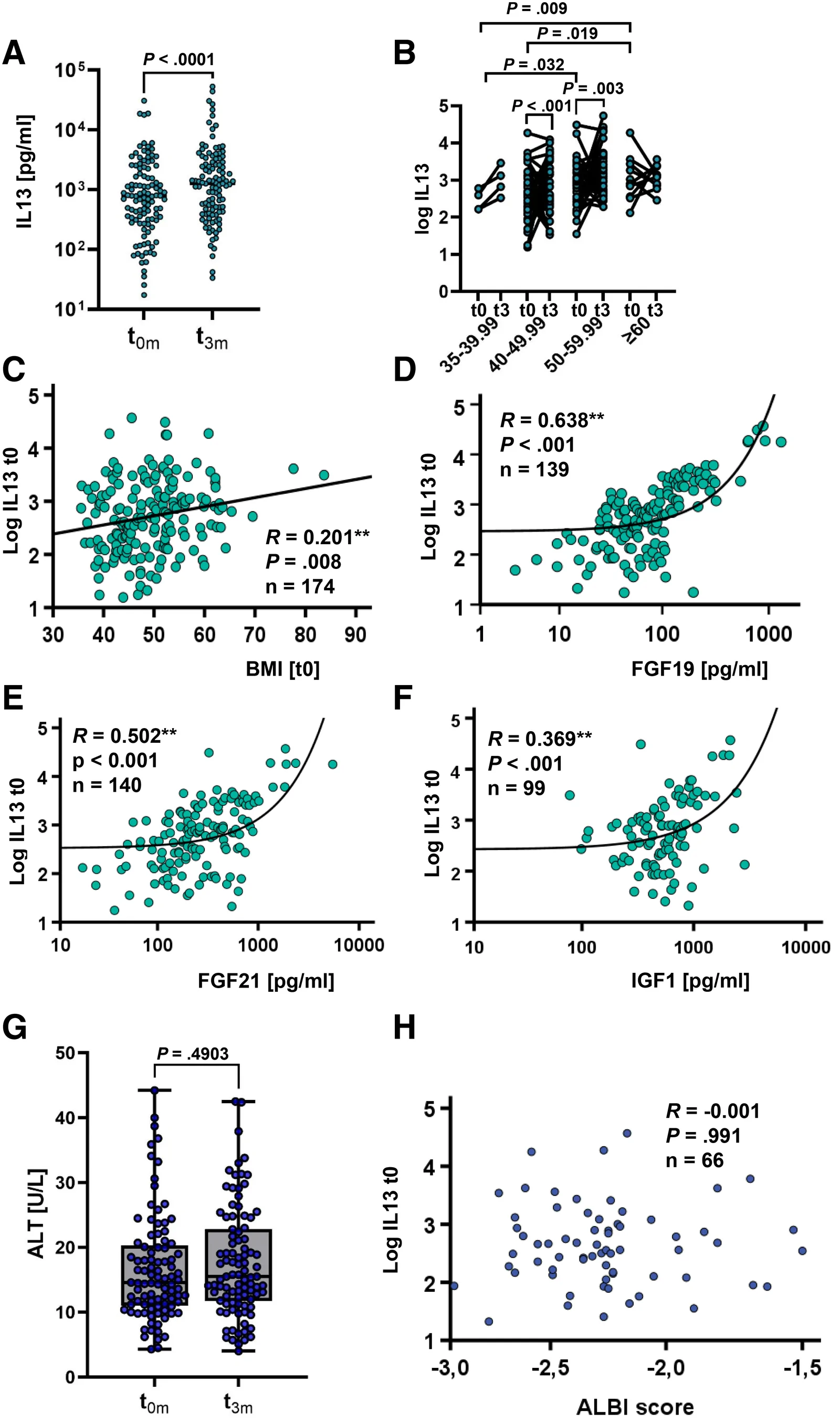
**Plasma IL13 concentration was positively associated with body weight loss in female patients with ≥class II obesity.** (*A*) IL13 plasma levels in patients with class II and III obesity were measured using enzyme-linked immunosorbent assay (ELISA). After a 3-month follow-up (t3), an increase in plasma IL13 was observed in patients with class II or higher obesity, regardless of whether they underwent a low-calorie diet or bariatric surgery (n = 108). Wilcoxon test comparing matched-pair samples with Spearman *Rho* = 0.7951. (*B*) Plasma IL13 concentrations increased from baseline to 3 months in patients with BMI 40–49.9 kg/m^2^ and 50–59.9 kg/m^2^. At baseline, IL13 levels were higher in patients with BMI ≥50 kg/m^2^ compared with those with BMI 35–39.9 kg/m^2^. Patients with BMI ≥60 kg/m^2^ demonstrated higher IL13 levels than those with BMI 40–49.9 kg/m^2^. Data are presented as mean ± standard deviation. Statistical significance was assessed using 2-way analysis of variance. (*C*) IL13 plasma levels were positively associated with the initial BMI in class II and III obese patients (bariatric surgery and low-calorie diet) (n = 174; t0). (*D* and *E*) Plasma FGF19 (n = 139) and FGF21 (n = 140) were positively associated with the IL13 plasma concentration in patients with class ≥II obesity (t0m). (*F*) IGF1 plasma concentration showed a positive association with plasma IL13 in patients with class ≥II obesity (t0; n = 99). (*C–F*) Curve fitting was performed to assess the correlation coefficients and the related *P* values (indicated in the figure, [*C–F*] Pearson’s *R*). (*G* and *H*) Assessment of IL13 in relation to liver inflammation, hepatocellular injury, and liver function in the female study cohort. (*G*) Serum ALT levels at baseline and after 3 months showed no significant differences (n = 113; Wilcoxon matched-pairs test, Spearman’s *Rho* = 0.2939). (*H*) No correlation was observed between baseline IL13 concentrations and the ALBI score (n = 66; Pearson’s *R* = −0.001). Data are presented as mean ± standard deviation; *P* values are indicated in the figures. ∗∗The correlation is significant at the 0.01 level (2-tailed).

Stratification by body mass index (BMI) showed increased IL13 levels from baseline to follow-up in the BMI 40–49.99 kg/m^2^ (*P* < .001) and 50–59.99 kg/m^2^ groups (*P* = .003) (Figure 1*B*). Patients with BMI ≥50 kg/m^2^ had higher IL13 levels than those with BMI 35 to 39.99 kg/m^2^ (*P* = .032; *P* = .009), and individuals with BMI ≥60 kg/m^2^ exceeded the BMI 40 to 49.99 kg/m^2^ group (*P* = .019). Baseline IL13 showed a positive association with BMI (Figure 1*C*).

Because fibroblast growth factor (FGF) 19 and FGF21 regulate metabolic homeostasis and hepatic lipid metabolism,^12^ we analyzed their association with IL13. Baseline IL13 was positively associated with circulating FGF19 and FGF21 (Figure 1*D* and *E*), although serum availability was limited for some patients. Because insulin resistance contributes to fatty liver disease^12^ and reduced insulin-like growth factor-1 (IGF1) levels are associated with increased insulin resistance,^13^ IGF1 was also evaluated. IL13 showed a positive association with IGF1 (Figure 1*F*).

Alanine aminotransferase (ALT) levels did not differ between baseline and follow-up (Figure 1*G*). Likewise, the Albumin-Bilirubin (ALBI) score^14^ showed no association with IL13 (Figure 1*H*).

Correlations between IL13 and FGF19, FGF21, and IGF1 were also observed in male patients (n = 64), where IL13 increased after intervention (Figure 2*A–C*), IL13 concentrations did not differ between sexes (Figure 2*D*), body weight decreased (Figure 2*E*), ALT levels remained unchanged (Figure 2*F*), and IL13 was positively associated with FGF19, FGF21, and IGF1 (Figure 2*G–I*). However, interpretation in male patients is limited by the smaller sample size and incomplete data availability across analyses.

**Figure 2:**
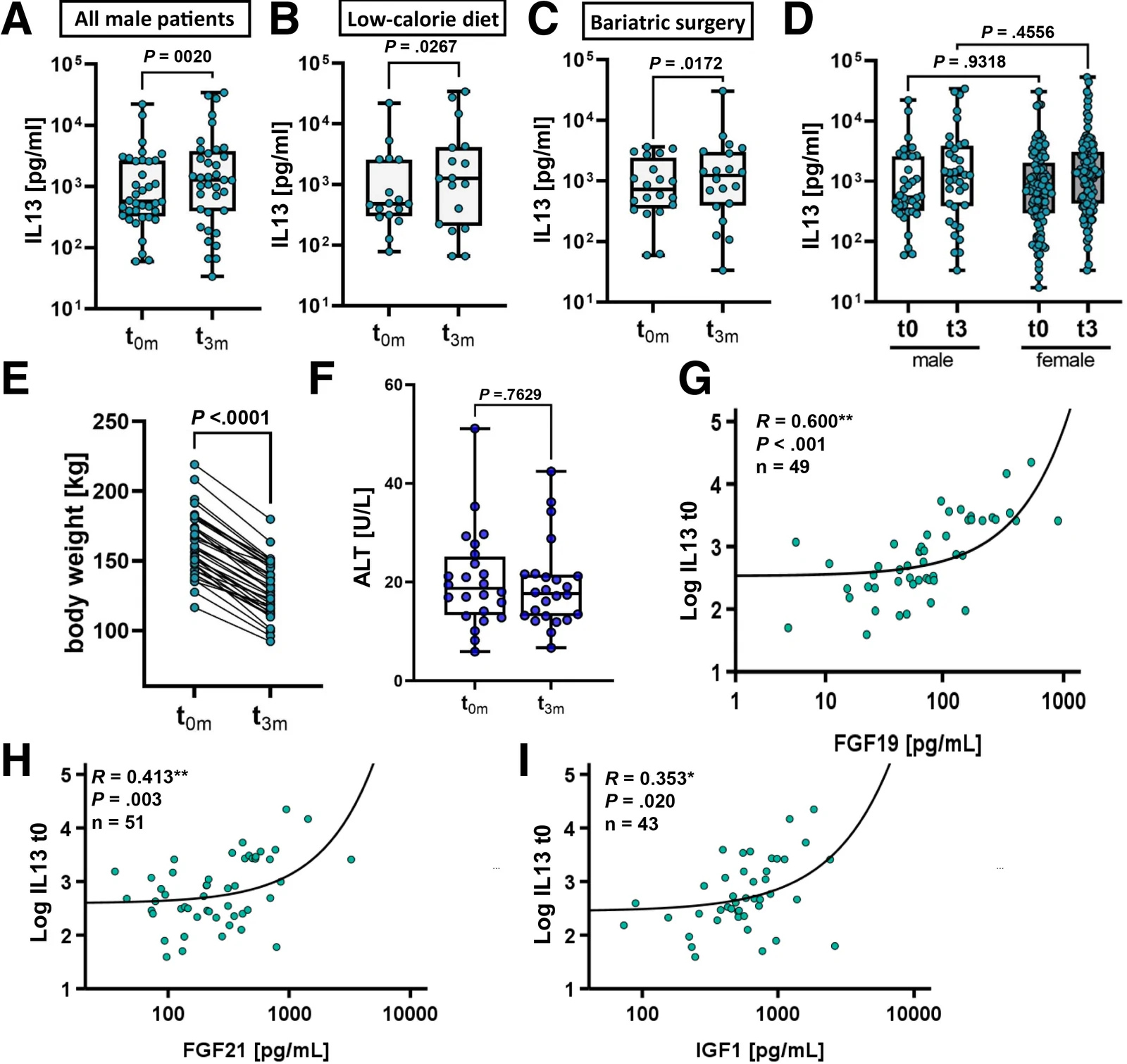
**IL13 plasma levels increase in male patients with severe obesity and were associated positively with FGF19, FGF21, and IGF1.** (*A−C*) IL13 plasma levels were measured at baseline (t0) and after 3 months (t3) in male patients with class ≥II obesity (n = 36). A significant increase in IL13 was observed in male patients following either a low-calorie diet (*B*; n = 17) or bariatric surgery (*C*; n = 19). Paired samples were compared using the Wilcoxon matched-pairs test; Spearman’s correlation coefficient: ρ = 0.8171 (*A*), *P* = .7627 (*B*), *P* = .9068 (*C*). (*D*) No significant differences in IL13 concentrations were detected between sexes (male group, n = 36; female group, n = 108) at baseline and follow-up (2-way analysis of variance). (*E*) A significant reduction in body weight was observed after 3 months of intervention, confirmed by the Wilcoxon test; Spearman’s ρ = 0.9238. (*F*) Serum ALT levels in the male study cohort at baseline and after 3 months. No significant differences were observed (n = 24; Wilcoxon matched-pairs test). Data are shown as mean ± standard deviation. (*G−I*) At baseline, IL13 concentrations were positively associated with FGF19 (n = 49), FGF21 (n = 51), and IGF1 levels (n = 43) in patients with class ≥II obesity. Pearson’s correlation coefficients (R), fitted regression curves, and corresponding *P* values are shown. ∗The correlation is significant at the .05 level (2-tailed). ∗∗The correlation is significant at the .01 level (2-tailed).

### The Hepatic Lipid Metabolism Was Dysregulated in *IL13*^*−/−*^ Mice

Disruptions in lipid metabolism are key drivers of hepatic steatosis.^15^ To delineate the physiological role of IL13 and to determine whether the absence of IL13 is sufficient to alter hepatic lipid metabolism under basal conditions, 8-week-old female *IL13*^*−/−*^ mice on normal chow were compared with wild-type (WT) controls. Body weight and serum ALT were similar between groups (Figure 3*A* and *B*). However, *IL13*^*−/−*^ mice showed elevated serum TGs (Figure 3C), whereas hepatic TGs and cholesterol remained unchanged (Figure 3*D*). Phosphatidylserine levels were increased in *IL13*^*−/−*^ mice liver tissue (Figure 3*D*).

**Figure 3:**
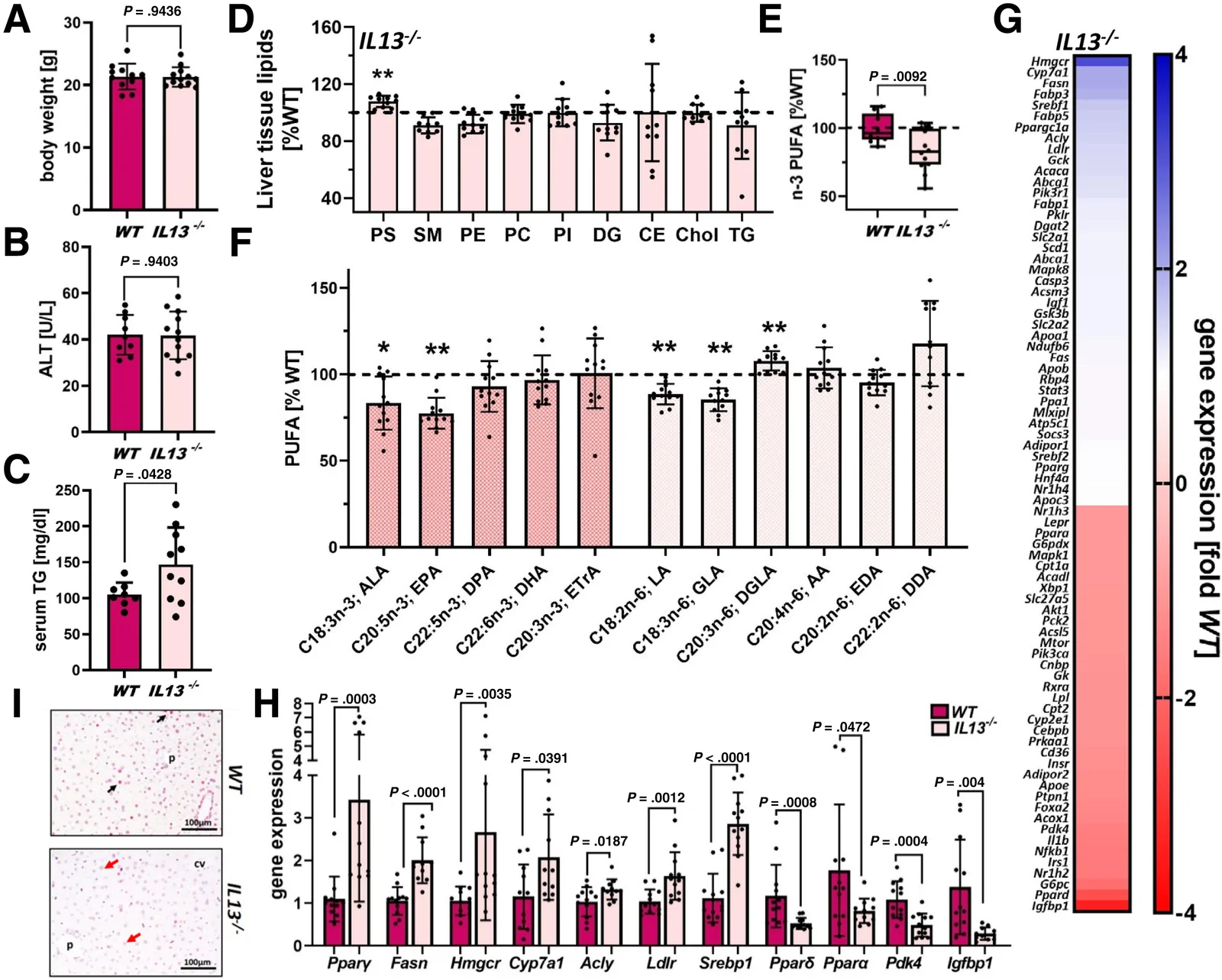
**The hepatic lipid metabolism was dysregulated in *IL13*^*−/−*^ mice.** (*A*) Body weight (n = 11–12) and (*B*) serum ALT (n = 9–12) remained unaffected in *IL13*^−/−^ mice. (*C*) Quantification of serum TG showed a significant increase in *IL13*^*−/−*^ mice compared with WT mice (n = 8–10). (*D*) The quantification of selected lipid species in hepatic tissue revealed an enhanced hepatic level of phosphatidylserine in *IL13*^*−/−*^ mice (n = 10–12). (*C* and *D*) Data represent the mean ± standard deviation (SD) from 2 technical replicates. (*E*) Hepatic n-3 PUFA was decreased in *IL13*^*−/−*^ compared with WT (n = 10–12). (*F*) Quantification of total hepatic PUFA in *IL13*^*−/−*^ mice compared with WT mice showed lower amounts of the n-3 PUFA α-linolenic acid (C18:3 c9,12,15; *P* = .0012) and EPA (C20:5 c5,8,11,14,17; *P* = .0493). Although the n-6 PUFA linoleic acid (C18:2; *P* =.0014) and γ-linolenic acid (C18:3 c6,9,12; *P* = .038) were lower in *IL13*^*−/−*^ mice, dihomo-γ-linolenic acid (C20:3 c8,11,14; *P* = .0088) was increased (n = 10–12). Each data point represents an individual animal (n = 10–12) from a single experiment. Mean ± SD is indicated. *Darker bars* indicate n-3 PUFAs; *lighter bars* indicate n-6-PUFAs. (*G*) RT fatty liver profiler PCR array analysis revealed marked dysregulation of hepatic genes involved in lipid metabolism in *IL13*^*−/−*^ mice. Representative array from a single experiment using complementary DNA (cDNA) of n = 10–12 animals as described under Methods. (*H*) Array results of hepatic tissue from *IL13*^*−/−*^ mice were confirmed by qRT-PCR analysis showing a dysregulation of genes involved in fatty acid β-oxidation, de novo lipogenesis (DNL), lipid uptake and transport, cholesterol synthesis, and glucose metabolism (N = 12). Data represent the mean ±SD from 3 independent experiments. Statistical significance was determined by Student *t* test and were indicated in the figure. PS, phosphatidylserine. (*I*) Immunohistochemistry staining revealed a reduction in nuclear PPARδ in liver tissue of *IL13*^*−/−*^ mice (*black arrows* indicate nuclear PPARδ staining in *red* in WT; *red arrows* indicate reduced nuclear PPARδ staining in *IL13*^*−/−*^ mice). A representative image is shown. CV, central vein; p, portal vein. Magnification, 200×.

Analysis of hepatic long-chain fatty acids revealed reduced polyunsaturated fatty acids (PUFAs) in *IL13* ^*−/−*^ mice (Figure 3*E*). Specifically, linoleic acid (C18:2) and γ-linolenic acid (C18:3 c6,9,12), substrates for arachidonic acid synthesis, were reduced, whereas dihomo-γ-linolenic acid (C20:3 c8,11,14) was increased (Figure 3*F*).^16^ α-Linolenic acid (C18:3 c9,12,15) and eicosapentaenoic acid (EPA) (C20:5 c5,8,11,14,17) were also decreased (Figure 3F). To assess transcriptional changes, lipid metabolism genes were analyzed using a real-time polymerase chain reaction (RT-PCR) array^17^ in pooled liver samples from *IL13*^*−/−*^ and WT mice (n = 12/group) (Figure 3*G*). Genes involved in lipid metabolism and transport, cholesterol metabolism, insulin signaling, and adipokine signaling showed >4-fold differential expression. Quantitative RT-PCR (qRT-PCR) confirmed increased expression of de novo lipogenesis genes (*Fasn, Srebp1, Acly*), cholesterol metabolism genes (*Hmgcr, Cyp7a1*), lipid transport (*Ldlr*), and *Pparγ* (Figure 3*H*). In contrast, genes involved in β-oxidation (*Pparα, Pparδ, Pdk4*) and glucose metabolism (*Igfbp1*) were downregulated (Figure 3*H*). Consistently, immunohistochemistry revealed reduced nuclear PPARδ in IL13-deficient liver tissue (Figure 3*I*). Together, these findings indicate that IL13 deficiency disrupts hepatic lipid homeostasis, promoting lipogenesis and reducing β-oxidation.

### Interleukin13 Reduced Hepatocellular Triglyceride Levels In Vitro

To examine whether IL13 directly affects hepatic lipid metabolism, primary mouse hepatocytes (PMHs) were treated with IL13 (12.5 ng/mL) for 24 hours in the presence or absence of oleic acid (OA; 1 mM) (Figure 4). This IL13 concentration reflects the upper range observed in patient plasma. Intracellular TG measurements showed that IL13 did not alter TG levels in nonsteatotic PMHs but significantly reduced TG accumulation under steatotic conditions (Figure 4*A*). Oil Red O staining confirmed reduced lipid accumulation in OA-treated hepatocytes exposed to IL13 (Figure 4*B* and *C*).

**Figure 4:**
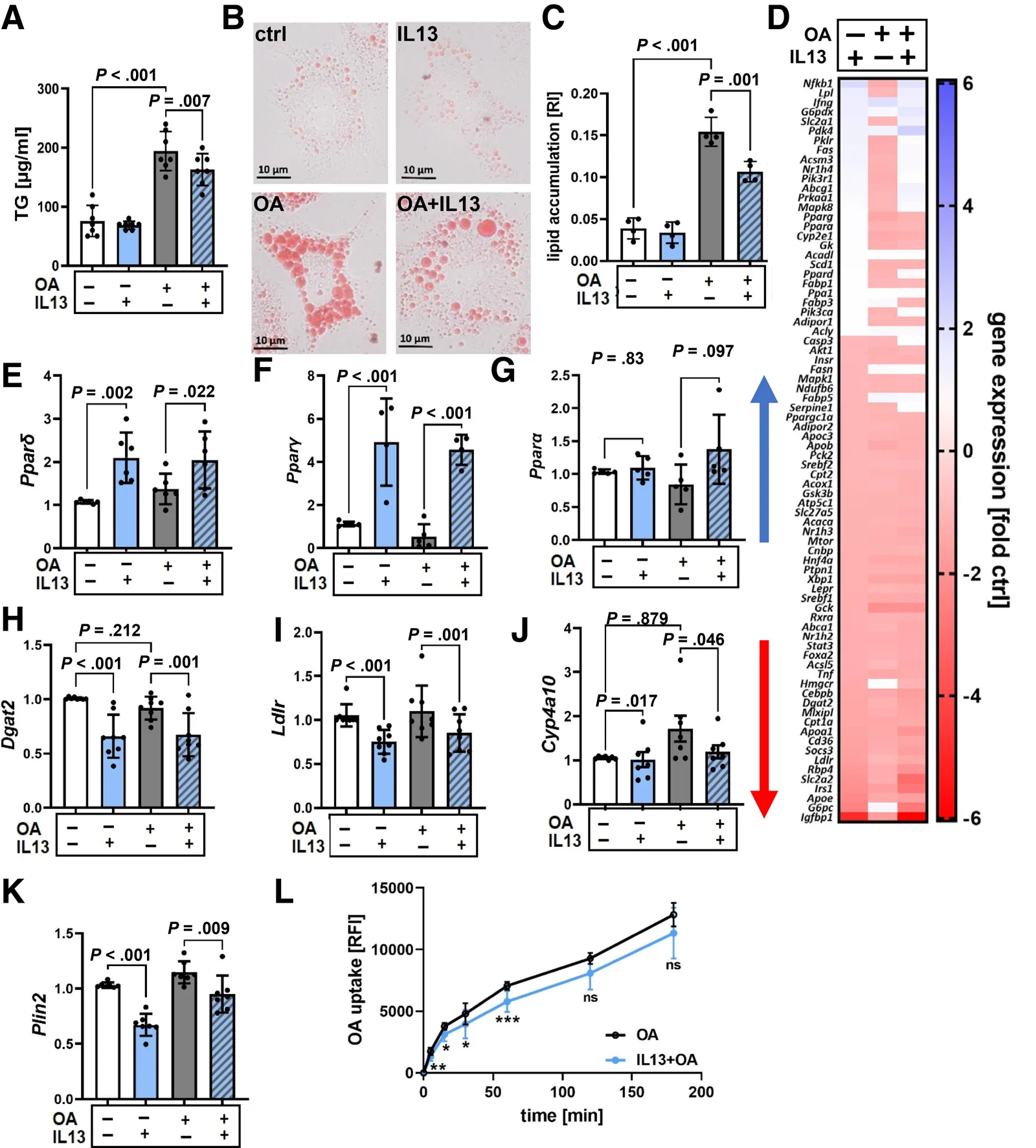
**IL13 ameliorates steatosis in vitro by lowering intrahepatocellular TG levels.** (*A*) PMHs were treated with 12.5 ng/mL IL13 or 12.5 ng/mL IL13 in the presence of 1 mM OA for 24 hours. Untreated murine hepatocytes were used as controls. The intracellular TG concentration was determined with the Diasys automated analysis system. IL13 lowered intracellular TG concentration in steatotic PMHs, whereas TG of nonsteatotic PMHs remained unaffected (N = 5 livers). Data represent mean ± standard deviation from 5 independent experiments (technical replicates), each performed under identical conditions. (*B* and *C*) Steatotic PMHs stained with Oil red O show reduced lipid accumulation in PMHs treated with IL13 (*lower right panel*; N = 4 livers, 4 technical replicates). The relative intensity (RI) of lipid accumulation in PMHs was quantified by ImageJ (*right side*). One representative image is shown. (*D*) RT fatty liver profiler PCR array analysis of IL13-treated PMHs or steatotic IL13-treated PMHs revealed a characteristic induction or inhibition of fat metabolism genes. Three groups were dedicated as x-fold vs untreated control cells. Data represent a single experiment with pooled complementary DNA of 7 livers as described under Methods. (*E–J*) qRT-PCR analysis of nonsteatotic and steatotic PMHs treated with OA and/or IL13 revealed upregulation of *Pparδ* (*E*)*, Pparγ* (*F*)*, and Pparα* (*G*) gene expression, whereas *Dgat2* (*H*), *Ldlr* (*I*), and *Cyp4a10* (*J*) gene expression levels were downregulated by IL13 (n = 5–8 livers). (*K*) Quantification of *Plin2* gene expression by qRT-PCR revealed downregulation of *Plin*2 by IL13 in non- and steatotic PMHs (n = 7 livers). Statistical group differences in (*A*, *C* and *E–K*) were obtained by 2-way analysis of variance. (*L*) Uptake of fluorescent-labelled OA was lower in the first 60 minutes. Data represent mean ± standard deviation from 4 (*L*) independent experiments (technical replicates), each performed under identical conditions. Statistical group differences were obtained by Student *t* test.

RT-PCR array analysis revealed modulation of genes involved in lipid metabolism, with downregulation of genes related to cholesterol metabolism and lipid transport (Figure 4*D*). IL13 increased expression of *Pparδ* (∼2-fold) and *Pparγ* (∼6-fold) in both nonsteatotic and steatotic PMHs, whereas *Pparα* (∼2-fold) and lipoprotein lipase (*Lpl*; ∼1.7-fold) were induced only under steatotic conditions (Figure 4*E–G*). Conversely, IL13 reduced the expression of *Dgat2* (∼0.7-fold), *Ldlr* (∼0.8-fold), and *Cyp4A10* (∼0.5-fold under OA stimulation) (Figure 4*H–J*). Although not included in the array, IL13 also decreased expression of the lipid droplet marker *Plin2* in both steatotic and nonsteatotic PMHs (Figure 4*K*). Finally, lipid uptake assays demonstrated reduced fatty acid uptake over time in IL13-treated hepatocytes (Figure 4*L*), indicating that IL13 limits lipid accumulation by attenuating hepatocellular lipid uptake.

### Interleukin13 Drives Hepatocellular JNK/c-JUN/FOSL2 Activation

Distinct transcription factor activator protein-1 (FOS/JUN) dimers exert protective effects in steatohepatitis,^9^ and IL13-dependent activation of FOSL2 has been described in epithelial cells.^18^ To assess whether IL13 regulates FOS/JUN family members in the liver, we analyzed hepatic tissue and primary hepatocytes. IL13 deficiency resulted in reduced hepatic *Fosl2* and *Jun* expression (Figure 5*A* and *B*), whereas IL13 stimulation induced *Fosl2* and *Jun* expression in both steatotic and nonsteatotic PMHs (Figure 5*C* and *D*).

**Figure 5:**
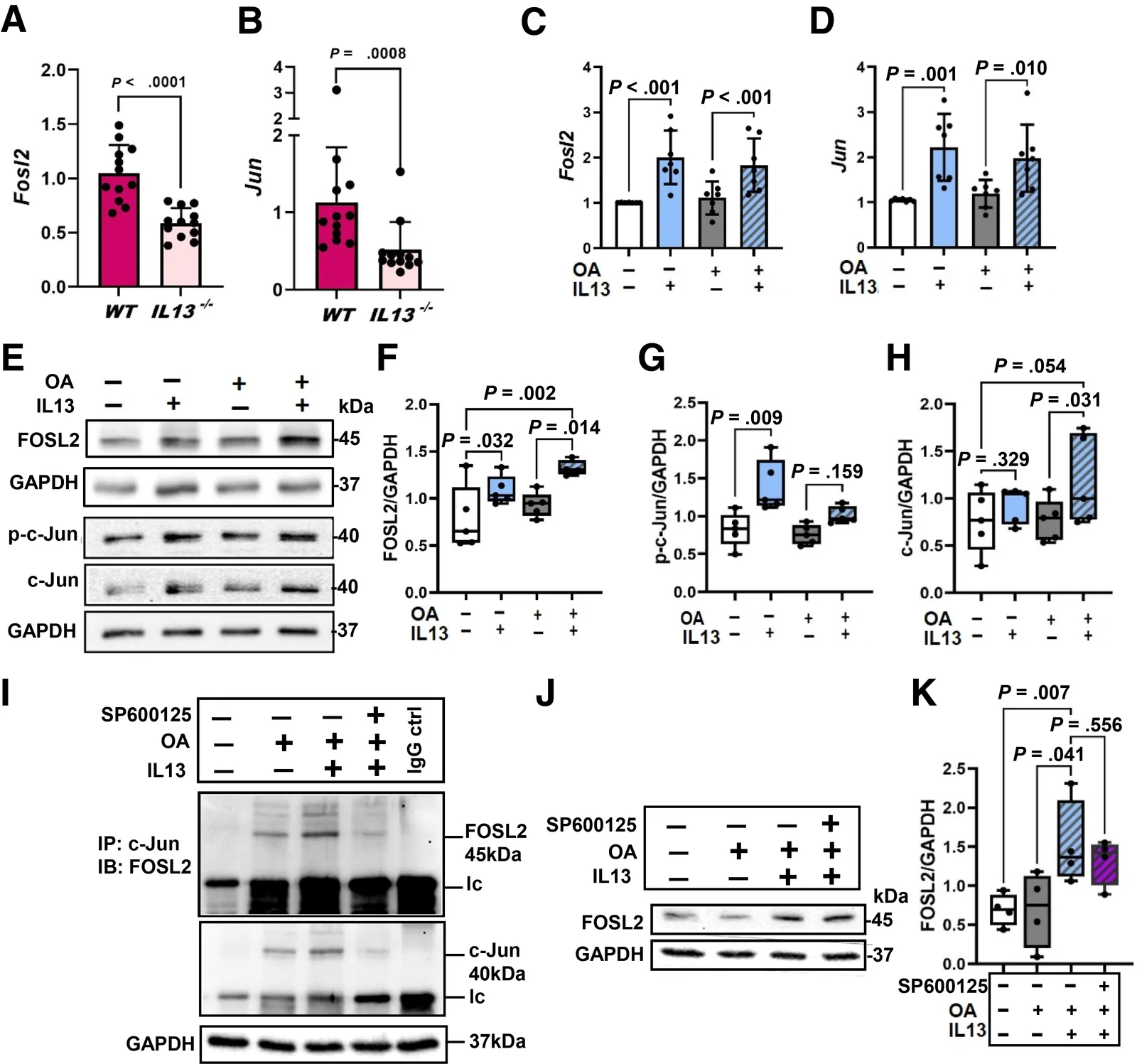
**Hepatocellular activation of c-JUN and FOSL2 was dependent on IL13-mediated JNK activation.** (*A* and *B*) Gene expression of *Fosl2* (*A*) and *Jun* (*B*) was downregulated in *IL13*^*−/−*^ mice (N = 12). Data represent the mean ± standard deviation from 3 independent experiments (technical replicates). (*C* and *D*) Gene expression analysis by qRT-PCR revealed that *Fosl2* (*C*) and *Jun* (*D*) were upregulated by IL13 in steatotic and nonsteatotic PMHs in vitro. (*E–H*) Western blot analysis demonstrated that IL13 treatment of both non-steatotic and steatotic PMHs in vitro increased FOSL2 protein expression (*E* and *F*; N = 5 livers). IL13 treatment led to activation of c-JUN as shown by c-JUN phosphorylation (*E* and *G*). c-JUN protein expression was also increased by IL13 in steatotic PMHs (*E* and *H*; N = 5 livers). (*I*) Immunoprecipitation of c-JUN and Western blotting for FOSL2 in IL13-treated PMHs revealed a c-JUN–FOSL2 dimer that was disrupted by JNK inhibition with SP600125. lc, light chain. (*J* and *K*) Western blot analysis revealed that protein levels of FOSL2 were not affected by JNK inhibition in steatotic PMHs (N = 4 livers). Data represent the mean ± standard deviation from at least 3 independent experiments, each performed under identical conditions (*A–D*, *F–H* and *K*). Relative quantification of Western blot analysis was performed by ImageJ. Representative Western blots are shown (*E* and *J*). Statistical group differences of WT compared to *IL13*^*−/−*^ mice were analyzed by Student *t* test (*A* and *B*), and group differences of PMHs were analyzed by 2-way analysis of variance (*C*, *D, F–H* and *K*).

Western blot analysis confirmed increased FOSL2 protein levels and phosphorylation of c-JUN following IL13 treatment in PMHs (Figure 5*E–H*). Immunoprecipitation further demonstrated formation of a c-JUN–FOSL2 heterodimer, which was disrupted by JNK inhibition with SP600125 (Figure 5*I*). Consistent with previous reports,^19^ JNK inhibition reduced c-JUN protein levels but did not affect FOSL2 protein expression (Figure 5*J* and *K*).

### Interleukin13 Reduced Intracellular Lipids via JNK/c-JUN/FOSL2 Axis

We next examined whether IL13-induced *Fosl2* expression mediates its effects on hepatic steatosis. Silencing *Il13ra1* by small interfering RNA (siRNA) in PMHs reversed the IL13-dependent reduction of lipid accumulation under steatotic conditions, as shown by Oil Red O staining (Figure 6*A* and *B*). *Il13ra1* expression itself was not altered by OA or IL13 (Figure 6*C*). Similarly, *Fosl2* knockdown abolished the lipid-lowering effect of IL13 and resulted in increased lipid accumulation despite IL13 stimulation (Figure 6*D* and *E*), whereas *Fosl2* expression was induced by OA and IL13 (Figure 6*F*). Consistently, FOSL2 overexpression reduced OA-induced lipid accumulation in PMHs (Figure 6*G–I*). To determine whether JNK-mediated activation of c-JUN–FOSL2 contributes to this effect, steatotic PMHs were treated with the JNK inhibitor SP600125. IL13 reduced TG SP600125 levels in OA-treated hepatocytes, but this effect was abolished by JNK inhibition, as shown by intracellular TG measurements (Figure 6*J*) and Oil Red O staining (Figure 6*K* and *L*). SP600125 alone did not affect lipid accumulation in OA-treated PMHs (Figure 6*J–L*).

**Figure 6:**
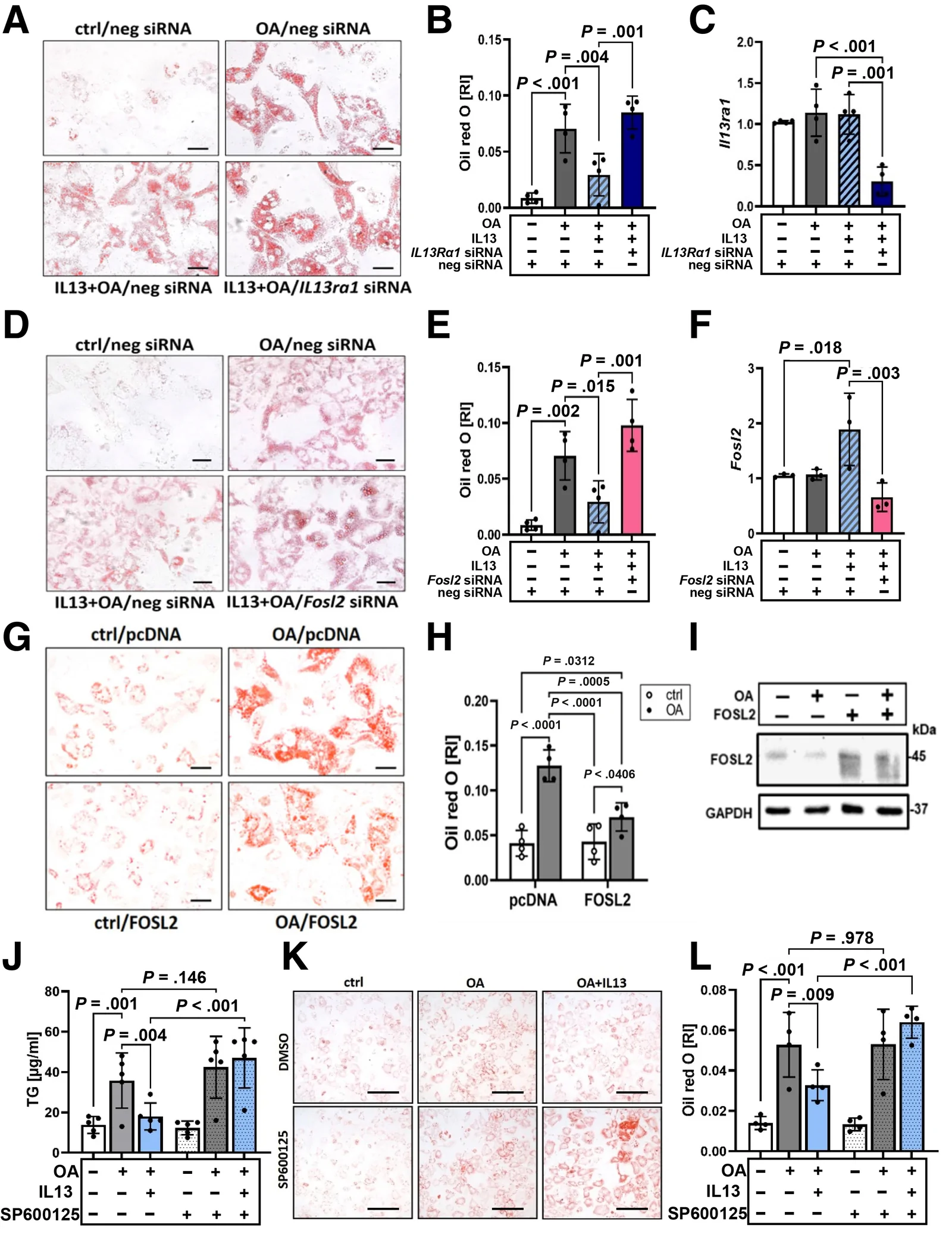
**IL13-induced downregulation of intracellular lipids was mediated via IL13Rα1–JNK–FOSL2 axis.** (*A–F*) Silencing of IL13Rα1 or FOSL2 by specific siRNAs abolished the IL13 effect of lipid reduction in steatotic PMHs. PMHs were transfected either with negative siRNA as a control, *IL13Ra1* siRNA (*A–C*), or *Fosl2* siRNA (*D–F*) and eventually treated with vehicle, IL13, OA, or OA+IL13 for 24 hours as indicated. qRT-PCR indicated a silencing of *IL13Ra1* (*C*) and *Fosl2* (*F*) in PMHs. The knockdown efficiency is demonstrated for *IL13ra1* knockdown (*C*) and for *Fosl2* knockdown (*F*) by siRNA. (*C*) siRNA-mediated knockdown reduced *IL13ra1* expression by 70% relative to the control level and by 77% compared with IL13+OA stimulation (*right panel*). (*F*) Knockdown of *Fosl2* by siRNA reduced *Fosl2* mRNA expression by 37% relative to control stimulation and by 65% relative to IL13+OA stimulation. (*A* and *D*) Oil red O staining of PMHs was performed to detect the lipid content. Relative quantification was determined by ImageJ analysis. Silencing of *IL13Ra1* (*A*) or *Fosl2* (*D*) reversed IL13-mediated decrease of hepatocellular lipids (N = 4 livers). (*G* and *H*) FOSL2 overexpression reduces hepatic lipid accumulation in PMHs. (*G*) Representative micrographs of PMHs transfected with a control vector (pcDNA) or overexpressing FOSL2. One representative image is shown. Oil Red O staining of steatotic PMHs shows reduced lipid accumulation in OA-treated cells overexpressing FOSL2 (*G*, *lower right panel*). The relative intensity (RI) of lipid accumulation was quantified using ImageJ and normalized to cell count (*H*). (*I*) FOSL2 expression levels were determined by Western blot analysis. Data represent mean ± standard deviation from 4 independent experiments performed using PMHs isolated from 3 livers (2 experiments derived from 2 liver). (*J*) IL13-induced reduction of hepatocellular TG was reversed by inhibition of JNK by SP600125 (N = 5 livers). (*K* and *L*) Visualization of lipid content was performed by Oil red O staining and quantified by ImageJ (N = 4 livers). Representative images of Oil red O staining are shown. Bars, 50 μm (*A, D* and *G*) and 200 μm (*K*). Data represent mean ± standard deviation from at least 3 independent experiments, each performed under identical conditions. Statistical differences were analyzed by analysis of variance.

### Interleukin13 Promotes Ketogenesis, Thereby Allevating Hepatic Steatosis

IL13-induced *Pparα* expression was reduced after *Fosl2* silencing (Figure 7*A* and *B*). As PPARα regulates fatty acid uptake, oxidation, and ketogenesis,^20^ we investigated whether IL13 improves fatty acid oxidation via FOSL2. FOSL2 overexpression enhanced PPAR reporter activity under basal and OA-stimulated conditions (Figure 7*C* and *D*), and a FOSL2-dependent effect was also observed for *Pparγ* (Figure 7*E*). RT-PCR array analysis of fatty acid metabolism genes revealed a distinct expression pattern in steatotic PMHs treated with IL13 (Figure 7*F*). IL13 counterbalanced the OA-induced upregulation of β-oxidation enzymes (*Oxct2a, Acat1, Acadsb*) and lipid transport genes (*Fabp5*), while restoring reduced expression of *Fabp1,2,4,* and *6* (Figure 7*F*). Silencing *Fosl2* reversed these IL13-dependent effects on β-oxidation and lipid transport (Figure 7*F*).

**Figure 7:**
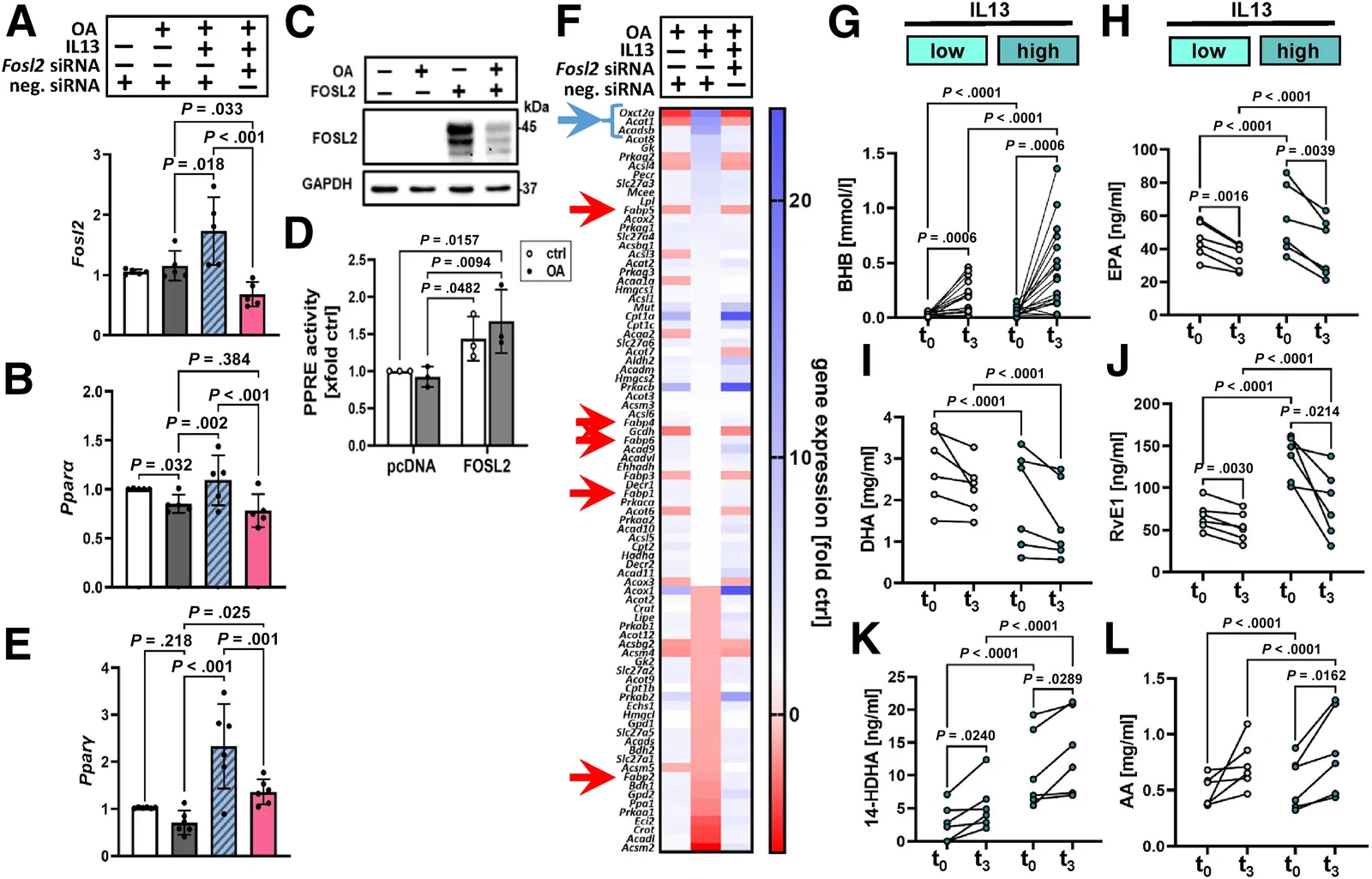
**IL13 reduced steatosis by FOSL2-mediated balancing of fatty acid oxidation and enhanced ketogenesis in MASLD.** (*A* and *B*) PMHs were transfected with negative siRNA (scrambled) as a control and *Fosl2* siRNA as indicated. Eventually PMHs were treated with vehicle control, OA, or OA+IL13. Gene expression was analyzed by qRT-PCR (n = 5–6 livers). (*A*) Transfection control for *Fosl2* knockdown. *Fosl2* gene expression was downregulated in OA+IL13 treated PMHs. Knockdown of *Fosl2* by siRNA reduced *Fosl2* mRNA expression by 25% relative to control stimulation and by 56% relative to IL13+OA stimulation. IL13 induced gene expression of *Pparα* (*B*). FOSL2 interacts with PPARs as shown by PPAR reporter assay. (*C*) Dual-luciferase PPAR reporter assay demonstrating that FOSL2 enhances PPAR-dependent transcriptional activity. Relative luciferase activity was normalized to control (Renilla luciferase activity). (*D*) Representative Western blot confirming FOSL2 overexpression. Data are derived from 3 independent experiments, each measured in triplicates. PPRE = PPAR Response Element. IL13-induced gene expression of *Pparɤ* (*E*) in steatotic PMHs were dependent on FOSL2 as shown by *Fosl2* silencing. Data represent mean ± standard deviation (SD) from 3 independent experiments. Statistical significance was obtained by 2-way analysis of variance. (*F*) RT profiler array for fatty acid metabolism was performed with *Fosl2* siRNA which revealed a disruption of IL13-mediated balancing of FA transport (*red arrows*), β-oxidation and ketogenesis (*blue arrow*) (*middle panel*) under silencing *Fosl2* by siRNA (*right panel*). Data represent a single experiment with pooled complementary DNA of 5 livers as described under Methods. (*G*) BHB was determined in plasma of 2 groups of patients with class III obesity (bariatric surgery group) with either low IL13 (<100 pg/mL) or high (>100 pg/mL) plasma levels at t0 (high IL13 n = 19; low IL13 n = 17). BHB was higher in patients with class III obesity with high IL13 plasma concentrations after 3-month follow-up. Data represent the mean ± SD from 2 technical replicates (t3 high, n = 16; low, n = 18). (*H–L*) Plasma of class III obesity study subjects (bariatric surgery group) was analyzed by LC-MS/MS technique to determine eicosanoid species EPA, docosahexaenoic acid (DHA), resolvin E1 (RvE1), 14-hydroxydocosahexaenoic acid (14-HDHA), and AA (n = 6 per group). Study subjects from the “high IL13 group” (>100 pg/mL) showed significant higher concentrations of EPA, RvE1, 14-HDHA and AA. The low IL13 group consisted of IL13 concentrations <100 pg/mL. Each data point represents an individual patient (n = 6) from a single experiment. Mean ± SD is indicated. Statistical significance was obtained by analysis of variance with pairwise comparisons.

Because β-oxidation generates acetyl-CoA that fuels ketogenesis,^21^ we examined circulating β-hydroxybutyrate (BHB) in patients with class III obesity after bariatric surgery. Patients with high IL13 (>100 pg/mL) showed higher BHB levels during weight loss than those with low IL13 (<100 pg/mL) (Figure 7*G*). Given that FOSL2 regulates EPA-responsive genes^22^ and omega-3 fatty acids reduce liver fat in metabolic dysfunction–associated steatotic liver disease (MASLD),^23^ we analyzed eicosanoid metabolism in patients with high or low IL13 using liquid chromatography–tandem mass spectrometry (LC-MS/MS) (Figure 7*H–L*). Although EPA and docosahexaenoic acid declined after surgery, patients with higher IL13 had higher EPA levels at baseline and follow-up (Figure 7*H* and *I*). The EPA-derived resolvin E1 and the docosahexaenoic acid–derived precursor 14-hydroxydocosahexaenoic acid were also increased in the high IL13 group (Figure 7*J* and *K*). Arachidonic acid levels increased after surgery in both groups but remained higher in patients with elevated IL13 (Figure 7*L*).

### Is FOS-Like Antigen 2 Associated With Amelioration of Steatosis in Obese Patients With Metabolic Dysfunction–Associated Steatotic Liver Disease?

Because IL13 reduced steatosis through FOSL2-dependent regulation of lipid uptake and β-oxidation, we examined whether FOSL2 is detectable in human MASLD. FOSL2 expression was analyzed in liver biopsies from patients with MASLD by immunohistochemistry, with obese patients without steatohepatitis serving as controls. Liver morphology was assessed by hematoxylin and eosin staining and fibrosis by Sirius Red/Fast Green (Figure 8*A*).

**Figure 8:**
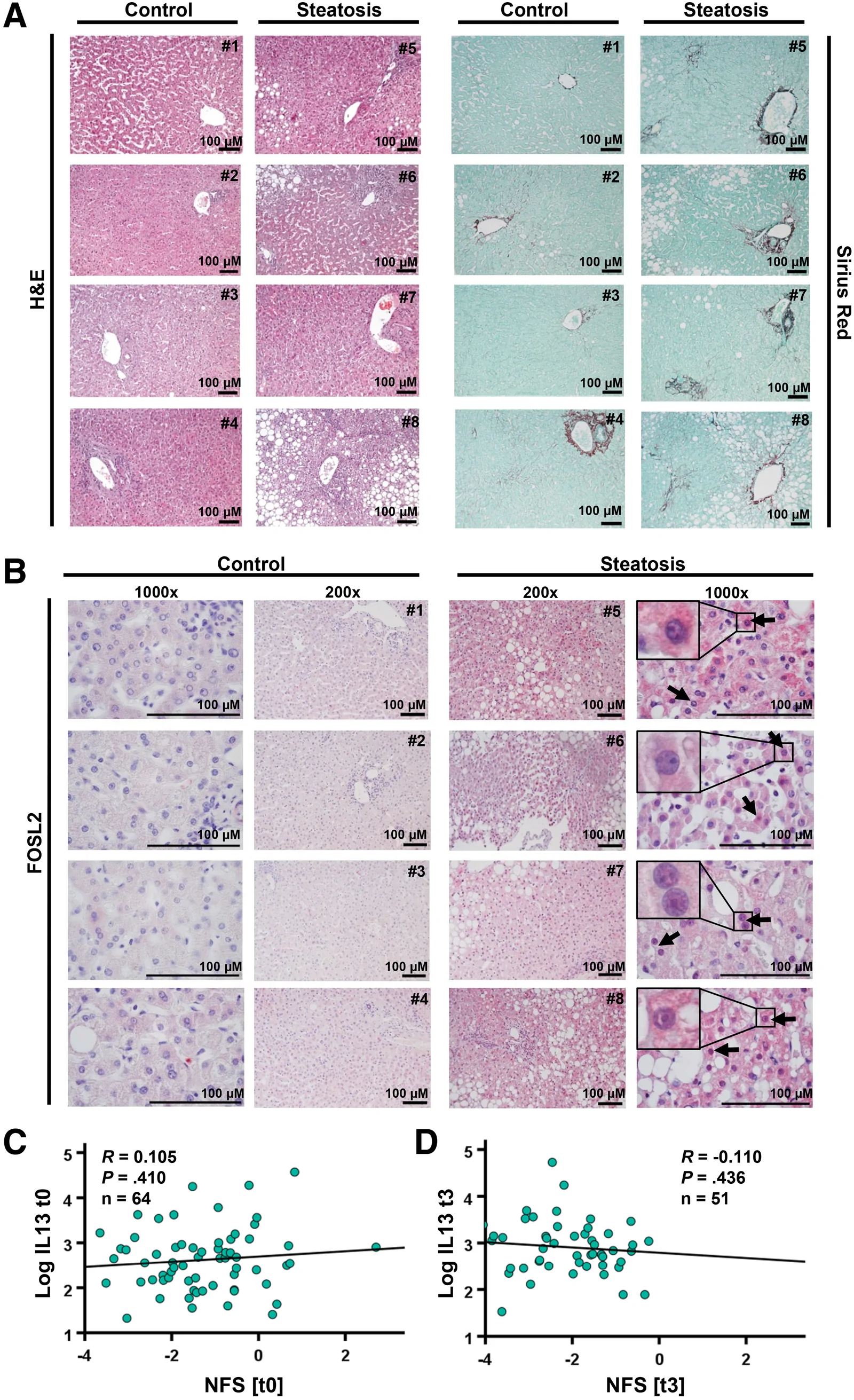
**Nuclear FOSL2 accumulation in patients with MASLD.** (*A*) Representative human liver biopsies from obese control individuals and female patients with MASLD (especially MASH) were stained with hematoxylin and eosin (H&E) (*left panel*) to assess liver tissue morphology and Sirius Red/Fast Green to visualize fibrosis (*right panel*). Patient IDs are indicated in the figure, with the NAS detailed as follows: The control group consisted of individuals with a BMI >40 kg/m^2^ (#1: S0A4F2, #2: S0A3F2, #3: S0A2F2, #4: S0A2F2). The steatosis and MASLD group included individuals with a BMI >50 kg/m^2^, with NAS as follows: #5: S1A2F2, #6: S2A2F2, #7: S2A3F2, #8: S3A2F2. (*B*) FOSL2 protein expression was determined by immunohistochemistry in human liver biopsies of female patients with MASLD (#5–8) and an obese control group (#1–4) serving as controls. NAS scores for all patients are mentioned under Methods. FOSL2 is visualized in *red*, whereas nuclei are counterstained in *blue*. When FOSL2 is localized in the nuclei, the overlap of *red* and *blue* results in a *dark violet* appearance (*black arrows*). In steatotic liver tissue, FOSL2 staining is more intense overall, with a notable presence in both the cytoplasm and nuclei, compared with the nonsteatotic controls. Representative images are shown at 200× (*A* and *B*) and 1000× magnification (*B*). (*C* and *D*) Plasma IL13 levels at baseline (t0) or after 3 months (t3) did not correlate with the NFS at baseline (*C*) or after 3 months (*D*), respectively.

Control biopsies (BMI >40 kg/m^2^) showed minimal steatosis (NAFLD Activity Score [NAS], S0), mild–moderate inflammation (NAS, A2–A4), and mild fibrosis (NAS, F2). In contrast, the steatosis and MASLD group (BMI >50 kg/m^2^) exhibited steatosis (NAS, S1–S3), predominantly in zone 3, with moderate to severe inflammation (NAS, A2–A3) and inflammatory cell infiltration around ballooned hepatocytes. Notably, nuclear FOSL2 staining was clearly increased in the steatosis and MASLD group compared with controls (Figure 8*B*), suggesting that FOSL2 induction may be associated with a compensatory response to hepatic steatosis. However, IL13 plasma concentrations did not correlate with the NAFLD Fibrosis Score (NFS) at baseline or after 3 months (Figure 8*C* and *D*).

## Discussion

We demonstrate that IL13 mitigates hepatocellular steatosis via the IL13Rα1–JNK-c-JUN–FOSL2 axis. IL13 suppressed lipogenic genes (*Dgat2*, *Ldlr*, *Cyp4A10*), induced *Pparα/δ/γ*, reduced fatty-acid uptake, and normalized steatosis-associated increases in transport and β-oxidation genes. Conversely, IL13 deficiency in mice shifted hepatic lipid metabolism towards enhanced lipogenesis and diminished β-oxidation, consistent with metabolic dysregulation.^8^ These experimental findings were paralleled clinically by elevated plasma IL13, BHB, and altered eicosanoids in patients with class II and III obesity.

Weight loss—dietary or surgical—was associated with increased circulating IL13 and was positively associated with BMI reduction. Given the high prevalence of MASLD in bariatric cohorts, this increase may reflect metabolic improvement.^24^ Although physiological IL13 levels are approximately 10 pg/mL,^25^ concentrations in our cohort showed wide variability and reached the nanogram range, comparable to levels reported in inflammatory and malignant conditions.^26^, ^27^, ^28^, ^29^, ^30^, ^31^, ^32^ Visceral adipose tissue, particularly group 2 innate lymphoid cells, represents a major source of IL13 in obesity.^33^ We believe that elevated IL13 in individuals with higher BMI likely reflects a compensatory response to increasing metabolic stress and tissue inflammation. Similar compensatory increases have been described for several cytokines and hormones during obesity, where elevated circulating concentrations do not necessarily translate into sufficient biological activity to prevent disease progression. Importantly, our human analysis is cross-sectional and therefore does not establish causality. In fact, previous studies have demonstrated that IL13 expression is increased in the adipose tissue of obese humans and high-fat diet–fed mice. Kwon et al identified adipocytes as a major source of IL13 and showed that proinflammatory cytokines such as tumor necrosis factor-α and IL1β induce IL13 expression, suggesting that IL13 represents a compensatory anti-inflammatory feedback response to obesity-associated inflammation rather than a causal driver of obesity. Therefore, elevated circulating IL13 in individuals with higher BMI seems to be compatible with a protective role of IL13 in limiting metabolic inflammation.^34^

IL13 was associated positively with FGF21 and FGF19, endocrine regulators of metabolic homeostasis with therapeutic promise in MASLD.^35^ As PPARα governs fatty-acid oxidation and fasting-induced FGF expression, the IL13–PPAR–FGF axis may attenuate lipid overload.^36^ The association between IL13 and IGF1 further implicates improved insulin sensitivity, aligning with established metabolic effects of IL4 and IL13 signaling.^37^

To delineate the physiological role of IL13 and to determine whether the absence of IL13 is sufficient to alter hepatic lipid metabolism under basal conditions, we used *IL13*^*−/−*^ mice. This model was not intended to recapitulate the clinical situation in patients but rather to investigate the physiological role of IL13 and to determine whether the absence of IL13 affects hepatic lipid metabolism under basal conditions. In IL13-deficient mice, normocaloric feeding induced de novo lipogenesis and cholesterol synthesis, accompanied by altered PPAR isoform expression (↑PPARγ; ↓PPARα/δ). *IL13*^*−/−*^ mice maintained on a normal diet did not develop overt hepatic steatosis, as reflected by unchanged body weight, histologic scores, serum ALT, cholesterol levels, and hepatic TG content. This observation is fully consistent with our experimental design and supports the notion that IL13 deficiency alone is not sufficient to induce steatotic liver disease. Importantly, the absence of a macroscopic steatotic phenotype does not preclude biologically relevant alterations at the transcriptional and signaling level, which may become functionally relevant under metabolic challenge. Although PPARγ exerts complex, context-dependent effects in the liver,^38^ IL13 induced all PPAR isoforms in steatotic hepatocytes together with FOSL2 and c-JUN, suggesting a coordinated transcriptional control. IL13 also increased *Pparδ* and *Lpl* expression, indicating improved lipid handling.^39^ Given that our clinical observations were derived from a cohort of individuals with obesity rather than patients with metabolic dysfunction–associated steatohepatitis (MASH), we intentionally restricted our mechanistic investigations to the direct effects of IL13 on hepatocytes. Addressing the role of IL13 in MASH would necessitate the development of a cell type–specific experimental model capable of disentangling hepatocyte-intrinsic effects from those mediated through other relevant cell types, including hepatic stellate cells and intestinal epithelial cells. The establishment and validation of such a sophisticated conditional mouse model would represent a substantial undertaking that lies well beyond the scope of the present work. Although fibrosis is the clinically most relevant endpoint in MASLD and MASH,^40^^,^^41^ the present work was designed to address early metabolic and inflammatory events. Hepatic steatosis therefore serves as an appropriate and sensitive readout of early disease pathophysiology within the scope of this study.

Lipidomic analyses revealed marked hypertriglyceridemia and altered phosphatidylserine in IL13-null mice, alongside reduced PUFAs (EPA, α- and γ-linolenic acids). Given that PUFAs activate PPARα and suppress de novo lipogenesis,^42^ their reduction supports a shift towards lipid accumulation*.* In vitro, IL13 limited lipid uptake and downregulated fatty acid binding proteins in a FOSL2-dependent manner, likely reducing intracellular fatty acid trafficking in steatotic hepatocytes.

Furthermore, IL13 modulated mitochondrial and peroxisomal β-oxidation gene expression and enhanced ketogenesis in individuals with high circulating IL13. Nuclear FOSL2 in steatotic livers suggests a compensatory response to lipid excess. However, given the context-dependent roles of JNK1 in steatohepatitis,^43^ the precise pathophysiological significance of the IL13Rα1–JNK–FOSL2 axis in MASLD remains to be defined.

### Limitations

As for the data from patients, we report only associations, which do not allow them to distinguish between cause and consequence. Not all clinical and biochemical parameters were available for every patient, which may have reduced the statistical power of certain analyses. We posit that IL13 may exert a dual pathogenic influence: first, by compromising intestinal epithelial barrier function, thereby facilitating the translocation of microbial products and amplifying hepatic innate immune activation^6^; and second, by directly enhancing fibrogenic responses within the liver through activation of mesenchymal effector cells.^44^ The convergence of gut-derived inflammatory cues^6^ and IL13-driven profibrotic signaling could thus represent a mechanistic axis that accelerates disease progression from metabolic injury to advanced fibroinflammatory remodeling. Our central hypothesis is that precise, organ- and cell type–specific modulation of the IL13 signaling pathway constitutes a rational and potentially transformative therapeutic strategy in hepatic steatosis rather than systemic cytokine application.

This study is the first to show that IL13 can directly reduce hepatic steatosis in primary hepatocytes and that IL13 deficiency disrupts hepatic lipid homeostasis, promoting lipogenesis and reducing β-oxidation in murine liver tissue. IL13 ameliorates fat metabolism by an IL13Rα1–JNK–FOSL2–dependent mechanism. Plasma analysis of obese patients as well as human liver biopsies from patients with hepatic steatosis extended our preclinical observations to the clinical setting.

## Materials and Methods

### Sex as a Biological Variable

Our study analyzed archived samples from both female and male participants enrolled in bariatric surgery studies. No relevant sex-specific differences affecting the study outcomes were observed within the analyzed data from the human cohorts. The animal experiments were primarily conducted in female mice because we cannot conclusively exclude sex-specific influences based on the mouse model. In addition, we confirmed the main finding of our work (decrease in *Fosl2* expression in the absence of IL13) in a cohort of male mice.

### Class II and Class III Obesity Study Subjects

Plasma samples were obtained from participants of the ROBS (Research in Obesity and Bariatric Surgery) cohort,^45^ a prospective, monocentric, observational study in patients with severe obesity treated at the University Hospital Giessen, Germany (approval number 101/14). All research was conducted in accordance with both the Declarations of Helsinki and Istanbul, and written consent was given in writing by all subjects. Plasma collected at baseline (t0) and 3-month follow-up (t3) was analyzed. Because each assay required a previously unthawed aliquot, sample numbers varied by availability, and not all patients had matched t0 and t3 samples.

Overall, 75% of samples were from female participants, and 25% were from male participants; due to small male participant numbers (especially in the low IL13 group), analyses focused primarily on female participants. IL13 was measured in women at t0 (N = 188) and t3 (N = 150), and in men at t0 (N = 64) and t3 (N = 40). Female participants had BMI ≥35 kg/m^2^ and were categorized per guidelines^46^: class II obesity (BMI, 35–39.99 kg/m^2^; 10.1%) and class III obesity (BMI ≥40 kg/m^2^; 89.1%), including morbid (BMI, 40–49.99 kg/m^2^; 43.6%), super-obesity (BMI, 50–59.99 kg/m^2^; 36.2%), and ultra-obesity (BMI ≥60 kg/m^2^; 10.1%). Interventions comprised low-calorie formula diet (37%) or bariatric surgery (63%) between January 2015 and April 2021. Type 2 diabetes was present in 16.3% (n = 36). Fibrosis risk was assessed using the NFS per guidelines^47^; among women with available data (n = 75), 50.7% had no fibrosis (NFS <−1.455), 43.7% had intermediate fibrosis (−1.455 to 0.675), and 5.6% had advanced fibrosis (>0.675). NFS ranged from −3.81 to 2.71 at baseline (mean, −1.31 ± 1.25) and from −5.55 to 0.33 at 3 months (mean, −2.21 ± 1.13). Exclusion criteria included pregnancy, endocrine disorders, untreated bulimia nervosa or binge eating disorder, illicit drug use, malignancy, severe psychiatric disease, and psychopathologic instability. All participants provided written informed consent. Baseline characteristics and liver parameters stratified by sex and IL13 are shown in Tables 1 and 2. Values below the detection limit (<11.7 pg/mL) were excluded from mean calculations in the female low IL13 group.

### Metabolic Dysfunction–Associated Steatotic Liver Disease Study Subjects

Model-derived findings were validated retrospectively in archived human liver tissue obtained during bariatric surgery. Liver biopsies were analyzed histologically to determine the presence of MASLD, especially MASH. Patients were excluded if they lacked informed consent, were younger than 18 years, had malignant disease, chronic inflammatory disorders, or were immunocompromised.

For immunohistochemical analysis, female patients with MASLD (steatosis grade 1–3) and non-MASLD controls (steatosis grade 0) were randomly selected (n = 4 per group). Control individuals had BMI >40 kg/m^2^ (NAS scores: S0A4F2, S0A3F2, S0A2F2, S0A2F2), whereas the MASLD group had BMI >50 kg/m^2^ (NAS scores: S1A2F2, S2A2F2, S2A3F2, S3A2F2). The study was approved by the ethics committee of Heidelberg University (approval number S-876/2021). All research was conducted in accordance with both the Declarations of Helsinki and Istanbul, and written consent was given in writing by all subjects.

### Scores for the Prediction of Fibrosis and Liver Function

NFS = −1.675 + 0.037 × age + 0.094 × BMI + 1.13 × impaired fasting glucose (yes = 1, no = 0) + 0.99 × aspartate aminotransferase (AST)/ALT − 0.013 × platelet count − 0.66 × albumin.^48^

ALBI = (log_10_ bilirubin × 0.66) + (albumin − 0.085).^14^

BMI-AST/ALT-Ratio-Diabetes Score: The score is calculated by adding points for each of the following conditions: BMI ≥ 28 kg/m^2^: 1 point; AST/ALT ratio ≥ 0.8: 2 points; diabetes mellitus: 1 point.^49^

Fibrosis-4 index = age × AST/(platelet count × √(ALT).^50^

### Animal Model

Female *IL13*^*−/−*^ knockout mice (BALB/c-ll13^tm2Anjm^) and WT controls were used as previously described.^6^^,^^51^ At 8 weeks of age, mice were anesthetized by isoflurane inhalation and sacrificed by cervical dislocation. Liver and serum samples were collected from 12 animals per group and stored for further analyses. At study onset, serum samples were available from n = 9 WT and n = 12 *IL13*^*−/−*^ mice, whereas body weight data were available for n = 11 WT and n = 12 *IL13*^*−/−*^ animals. Reduced sample numbers in certain analyses were due to limited serum availability and exclusion of statistical outliers using Grubbs’ test. No significant differences in histologic scores between WT and *IL13*^*−/−*^ mice were observed.^6^ All animal experiments were conducted in accordance with the European Convention for the Protection of Vertebrate Animals used for Experimental and Other Scientific Purposes (ETS No. 123) and approved by the Regional Council Giessen (V54-19c 20 15 h 01 GI20/10 No. A29/2013 and No. 128/2014).

### Cell Culture Experiments

PMHs were isolated from female C57BL/6J mice aged 4–8 months using gentleMACS Perfusion Technology (Miltenyi) as previously described.^52^

Hepatocytes were seeded on collagen I-coated culture dishes and maintained in Hepatozyme (Gibco) supplemented with 10% fetal calf serum (FCS; Anprotec), 1% L-glutamin (Anprotec), and 1% penicillin–streptomycin (Anprotec) for 24 hours before stimulation.

PMHs were stimulated with 1 mM OA (Sigma), 12.5 ng/mL recombinant IL13 (Peprotech), or vehicle control (2% FCS) in Dulbecco’s Modified Eagle Medium (DMEM; Anprotec) containing 1% penicillin–streptomycin for 24 hours. For knockdown experiments, hepatocytes were transfected with siRNA targeting *Fosl2* or *IL13Rα1*, or with negative control siRNA (Ambion), using Lipofectamine 2000 (Invitrogen) 48 hours prior to stimulation. Knockdown efficiency was confirmed by qRT-PCR. For JNK inhibition experiments, PMHs were pretreated with 10 μM SP600125 (Merck) or vehicle control dimethyl sulfoxide (Roth) for 30 minutes before stimulation with OA and/or IL13. Each hepatocyte experiment was performed at least 3 times using cells from independent animals.

### Targeted Metabolomics: Eicosanoid Extraction and Mass Spectrometric Analysis via Liquid Chromatography–Tandem Mass Spectrometry

Eicosanoids were extracted from liver lysates and human plasma and analyzed by high-pressure LC-MS/MS as previously described.^53^ Briefly, 500 μL of human plasma samples were spiked with internal standard solution and treated with 1 μL of 10 mg/mL SW033291 (15-PGDH-Inhibitor, Cayman), 1 μL 0.1% butylated hydroxytoluene (Sigma) solution, 1 μL 12% EDTA (Fluka) solution. Subsequently, 1 mL 50% acetonitrile (Sigma-Aldrich) and 50% methanol (Sigma-Aldrich) were added for protein precipitation at 0 °C for 15 minutes. Precipitates were pelleted by centrifugation at 4 °C for 15 minutes (4450RCF). The supernatant was decanted and filled with pure water to 10-mL volume. The samples were prepared and extracted by solid-phase extraction as described previously.^54^ Specifically, the 1100 Series capillary LC system (Agilent Technologies Deutschland) was employed for analyzing the plasma of female patients with class III obesity (BMI >40 kg/m^2^) who underwent bariatric surgery. Eicosanoid metabolites were characterized by the following extracted ion–chromatogram method as described previously.^53^ For MS analysis, plasma samples were analyzed using the Daltonik amaZon SL (Bruker Corporation). Characteristic extracted ion chromatograms were utilized for both the identification and quantification of eicosanoids. The MS raw data were analyzed using Compass DataAnalysis Version 5.3 as previously described.^54^

### Immunohistochemistry

Immunohistochemical detections were performed as described previously.^55^ Formalin-fixed, paraffin-embedded human liver specimens were stained with a FOSL2 antibody (1:250, GeneTex, #GTX64443). The same procedure was used to stain formalin-fixed, paraffin-embedded mouse liver from *IL13*^*−/−*^ and WT specimens with a PPARδ antibody (1:1000, Novus).

### Histologic and Cell Staining

Human liver sections were stained with hematoxylin (Roth) and eosin (Shandon) for morphologic evaluation. Fibrosis was assessed using Sirius Red (Polysciences, Inc) and Fast Green (Roth).

For lipid staining, PMHs grown on coverslips were treated with OA for 24 hours, fixed in 5% paraformaldehyde (ICN Biochemical) and stained with Oil Red O (Serva). Coverslips were washed with 60% isopropanol (Sigma-Aldrich), rinsed with distilled water, and mounted using Fluoromount (Sigma-Aldrich). Images were acquired from randomly selected fields, and the staining intensity was quantified using ImageJ software.

### Gene Expression Analysis

RNA isolation, reverse transcription, and qPCR were performed as described previously.^55^ Primers were obtained from Microsynth. Relative gene expression was calculated using the ΔΔCt method.^17^ Cycle threshold (Ct) values greater than 35 were excluded to ensure reliable detection.

RT fatty liver and fatty acid metabolism profiler arrays were performed according to the manufacturer’s protocol (Qiagen). RNA from PMHs (N = 7 for fatty liver array; N = 5 for fatty acid metabolism array) and mouse liver tissue (N = 12) was pooled within groups, converted to complementary DNA, and analyzed using the array platform. Ct values were normalized to 5 housekeeping genes. Genes with Ct values >35 or Ct >30 in both control and treated samples were excluded due to unreliable expression levels.

### Western Blot Analysis

Western blotting was performed as described previously.^56^ Overexpression of FOSL2 plasmid was determined in HepG2 by a polyclonal FOSL2 antibody (1:1000, PA5-99459, Invitrogen) and for PMHs by the anti-rabbit FOSL2 antibody (1:1000, HPA004817, Merck).

### Immunoprecipitation

Protein interactions were analyzed by immunoprecipitation using the Capturem IP & Co-IP Kit (Takara). PMHs (1 × 10^6^ cells) were lysed and incubated with anti-mouse c-JUN antibody (1:100, clone L70B11, CST#2315, Cell Signaling Technology). Immunoprecipitated proteins were analyzed by Western blot using an anti-rabbit FOSL2 antibody (HPA004817, Sigma-Aldrich).

### Measurement of Triglycerides, Cholesterol, β-Hydroxybutyrate, and Alanine Aminotransferase

TGs and cholesterol were measured in serum from *IL13*^*−/−*^ and WT mice and in hepatocyte lysates using an automated analyzer (respons910, DiaSys). BHB was measured in plasma samples from female study participants, and ALT was analyzed in plasma samples from both sexes using the same system.

### Enzyme-Linked Immunosorbent Assays

Venous blood samples were collected in citrate monovettes (Sarstedt), and plasma was obtained by centrifugation. Circulating IL13 FGF19, FGF21, and IGF1 were quantified using enzyme-linked immunosorbent assays (Biotechne).

All measurements were performed in technical duplicates. Samples with intraduplicate variation greater than 20% were repeated. To improve detection of low IL13 concentrations, the standard curve was extended down to 11.7 pg/mL. For undiluted plasma samples analyzed below 98.3 pg/mL, blank and capture antibodies were prepared in phosphate-buffered saline to minimize albumin interference.

### Lipid Uptake Assay

PMHs were seeded in black 96-well plates (Greiner Bio-One) and stimulated with 50 mM TopFluor OA (Sigma/Merck KGaA) in the presence or absence of IL13. Fluorescence was measured at 0, 5, 15, 30, 60, 120, and 180 minutes using a microplate reader (Tecan Infinite Pro 200) at excitation 470 nm and emission 507 nm.

### Analysis of Fatty Acid Composition by Gas Chromatography

The gas chromatography measurement was carried out once with 12 animals per group. The analysis of the hepatic fatty acid composition of 12 animals per group was performed using a Perkin Elmer Clarus 580 gas chromatograph with flame ionization detection. Initially, an aliquot of 35 mg liver was shaken in the dark for 18 hours at ambient room temperature with a mixture of n-hexane and isopropanol (3:2 (v/v)) containing C19:0 (10.59 mg/mL) as an internal standard. Samples were then centrifuged (1200 × *g*, 10 minutes, 15 °C). One hundred fifty microliters of the supernatant was evaporated with a N_2_ stream at 37 °C. Fatty acids were subsequently transmethylated using 0.2 M trimethylsulfonium hydroxide solution in methanol (Macherey-Nagel). The resulting fatty acid methyl esters (FAMEs) were separated by a gas chromatograph with flame ionization detection system equipped with a polar capillary column (Zebron, 30 m, 0.25 m internal diameter, 0.25 mm film thickness; Phenomenex Ltd) and a flame ionization detector. The injection volume was 1 μL with a 1:20 split, and the carrier gas was helium with a flow rate of 3.9 mL/min at the beginning. The following temperature program was used: 2 minutes at 100 °C, then the temperature was increased at a rate of 3 °C per minute until 240 °C was reached, and then the temperature was held at 240 °C for 5 minutes. A commercial standard mixture (Supelco 37-component FAME-Mix No 18919) was used to identify the individual FAME by comparing the retention times of the standards with those of the sample. Evaluation of peak areas was performed with the EZChrom Elite Software (Agilent). For qualitative evaluation in terms of percentage area, FAMEs were calculated as a percentage of the total peak area. Amounts of individual FAMEs were quantified by using C19:0 as an internal standard.

### High-Performance Thin-Layer Chromatography of Lipids

High-performance thin-layer chromatography was performed in duplicate with slight modifications,^57^ using 12 animals per group. Briefly, liver tissue (50 mg) was extracted with n-hexane and 2-propanol 3:2 (V/V) for 1 hour and centrifuged, and the supernatant was dried under nitrogen gas. The residue was reconstituted in chloroform and methanol 2:1 (V/V) and analyzed as 4-mm bands (5 μL/band for polar lipids and 7.5 μL/band for nonpolar lipids) using respective calibration standards. Lipid separation was achieved on high-performance thin-layer chromatography silica gel 60 F254 MS grade plates with specific solvent systems: chloroform, methanol, ammonia, and water 30:17:2:1 (V/V/V) up to 75 mm for polar lipids and n-hexane, diethyl ether, and glacial acetic acid 43:7:1 (V/V/V) up to 65 mm for nonpolar lipids (previously chamber saturation with filter paper for 20 minutes along with plate activation for 10 minutes at 33% relative humidity). Quantification of the polar and nonpolar lipids was performed by fluorescence measurement at 366 and >400 nm (TLC Scanner 4, mercury lamp, measurement slit 6 mm × 0.3 mm, scanning speed 10 mm/s, data resolution 50 μm) after derivatization via the primuline reagent and drying for 5 minutes. Quantification of phosphatidylserine was performed by absorption measurement at 577 mm after specific derivatization via the ninhydrin reagent (1.5 mL, 8 g ninhydrin in 10 mL ethanol and glacial acetic acid 22:3 [V/V], blue nozzle, spraying level 3; TLC Plate Heater, 105 °C, 5 minutes) to distinguish from coeluted phosphatidylinositol.

### Dual-Luciferase Reporter Assay

HepG2 cells (CLS #330198; ordered 2019; cryostocks ≤10 passages) were cultured in DMEM with 10% FCS and 1% penicillin–streptomycin at 37 °C and 5% CO_2_ (Anprotec). Cells (4 × 10^4^/well) were seeded in 96-well plates and transfected with 0.1 μg FOSL2 expression plasmid (https://doi.org/10.1073/pnas.95.8.4333, Addgene plasmid #187907) or empty vector pcDNA3.1-Flag (Addgene plasmid #208051; RRID: Addgene_208051) together with the PPRE × 3-TK-luc reporter plasmid (https://doi.org/10.1073/pnas.95.8.4333, Addgene plasmid #1015) and pGL4 [hRluc/TK] (0.1 μg; Promega). After 48 hours, cells were treated with 1 mM OA or control medium (DMEM with 2% FCS) for 24 hours. Luciferase activity was measured using the Dual-Luciferase Reporter Assay System (Promega, E1910), normalized to Renilla luciferase, and recorded with a Tecan microplate reader (Tecan Infinite Pro 200).

### Statistical Analysis

Normal distribution was assessed using Kolmogorov–Smirnov tests or Q-Q plots.^58^ Correlations in the obesity cohort were analyzed using Pearson correlation. For certain analyses, data were logarithmically transformed (log10) to approximate normal distributions.

For BHB and eicosanoid analyses, plasma samples were stratified into low IL13 (<100 pg/mL or undetectable) and high IL13 (>100 pg/mL) groups. Comparisons between WT and knockout mice were performed using the Student *t* test. PMH experiments were analyzed using 2-way analysis of variance with repeated measures implemented as a mixed-effects model. Pairwise comparisons of estimated marginal means were performed using the least significant difference test. Densitometric data are presented as means with 95% confidence intervals. Statistical analyses were performed using SPSS version 26.0 and GraphPad Prism version 9.5.1.

## Declaration of Generative AI and AI-Assisted Technologies in the Manuscript Preparation Process

During the preparation of this work, the authors used Jenni AI and DEEPL in order to enhance the English phrasing. After using this tool/service, the authors reviewed and edited the content as needed and take full responsibility for the content of the published article.

## Supplementary Material

Supplementary Material

Extended PDF
