# Supplementary material for: Interleukin13 Ameliorates Hepatocellular Steatosis via IL13Rα1/JNK/FOSL2 Signaling Pathway
Source: Cell Mol Gastroenterol Hepatol. 2026 Sep 3;20(12):101879. doi: 10.1016/j.jcmgh.2026.101879 (PMC13678019; doi:10.1016/j.jcmgh.2026.101879)
Supplement: Supplementary file 1 [file mmc1.pdf]

Supplementary Westernblots

Figure 5 E

FOSL2 exp. nr. 1 (representative)+2

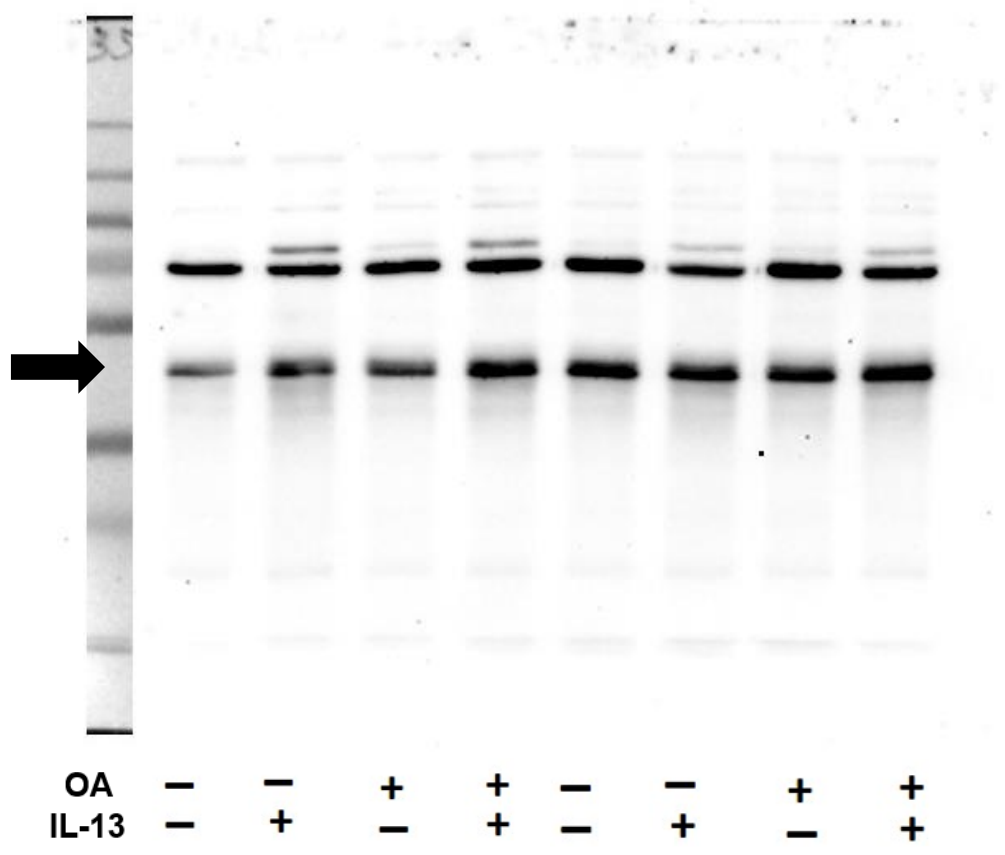

GAPDH exp. nr. 1 (representative) +2

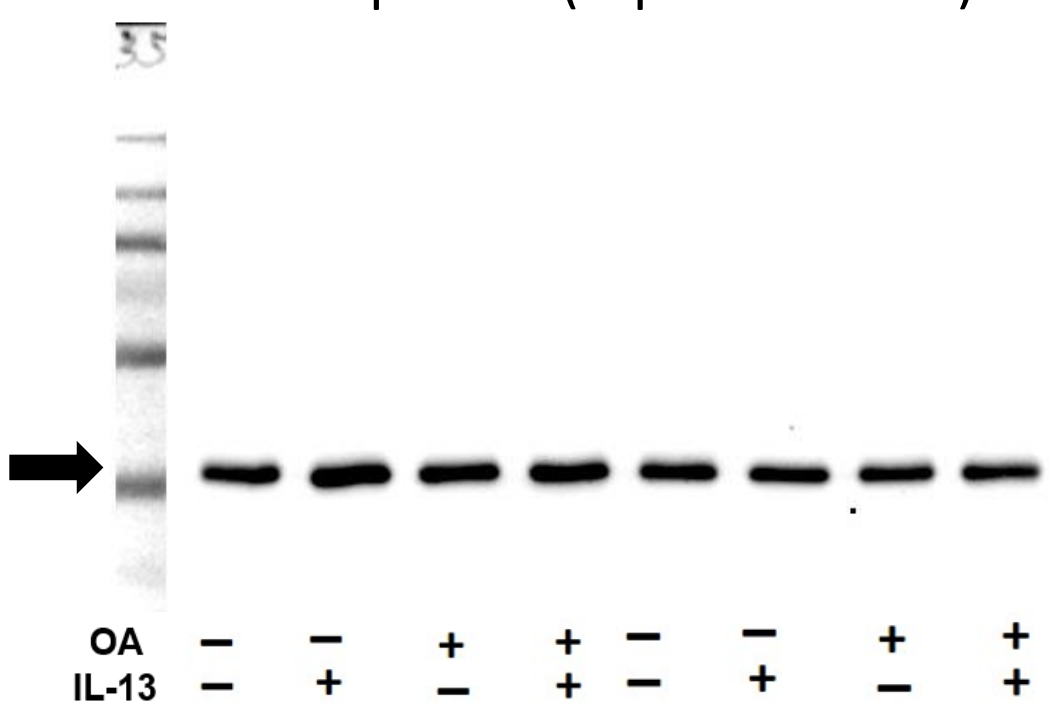

FOSL2 exp. nr. 3+4

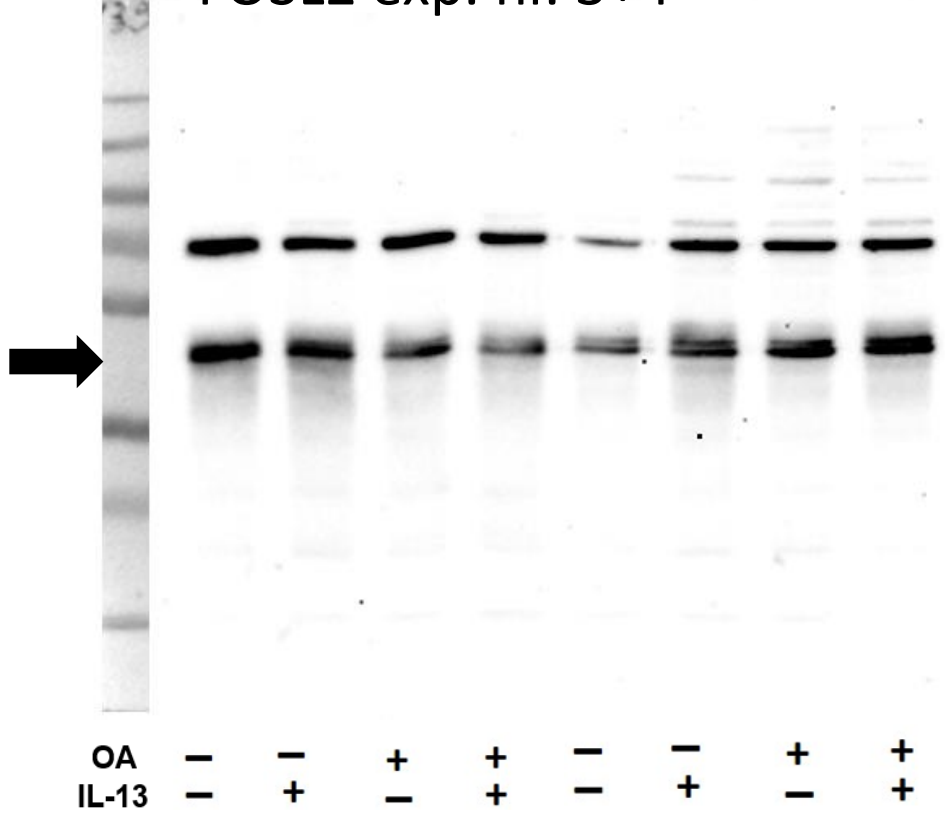

GAPDH exp. nr. 3+4

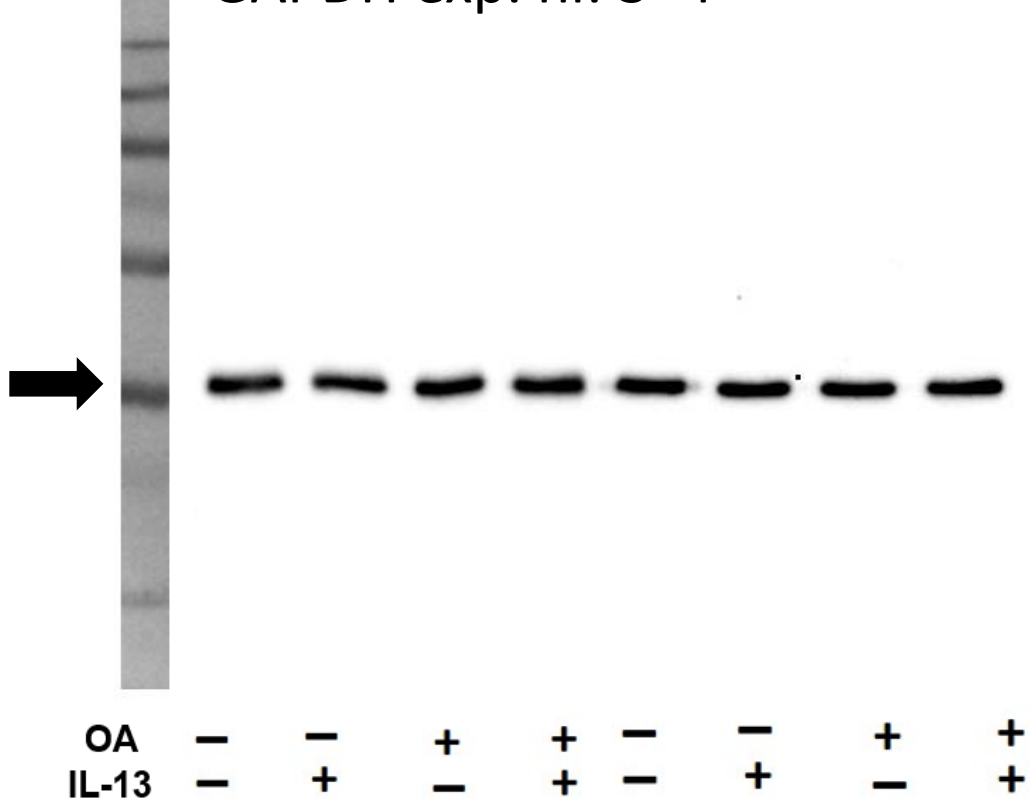

FOSL2 exp. nr. 5

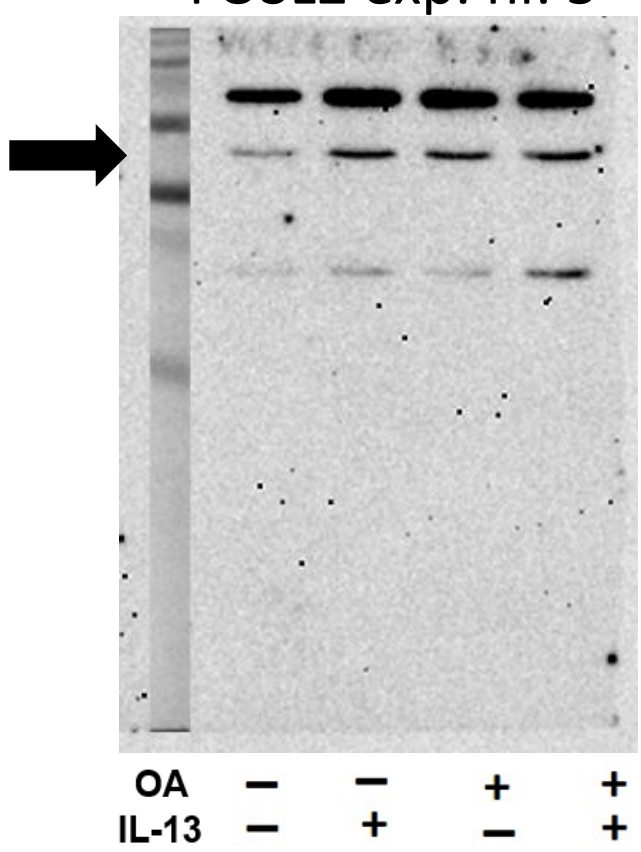

GAPDH exp. nr. 5

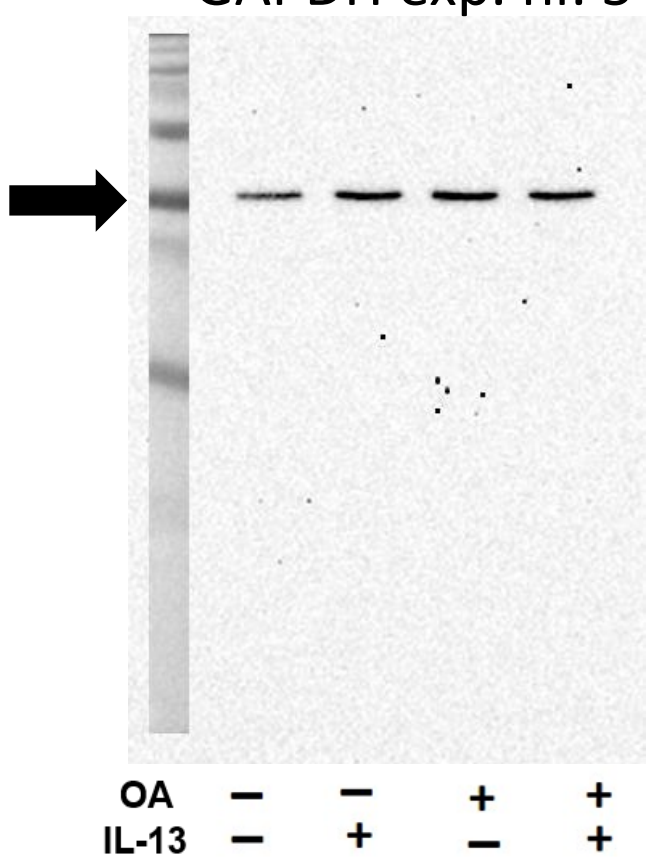

Figure 5 I

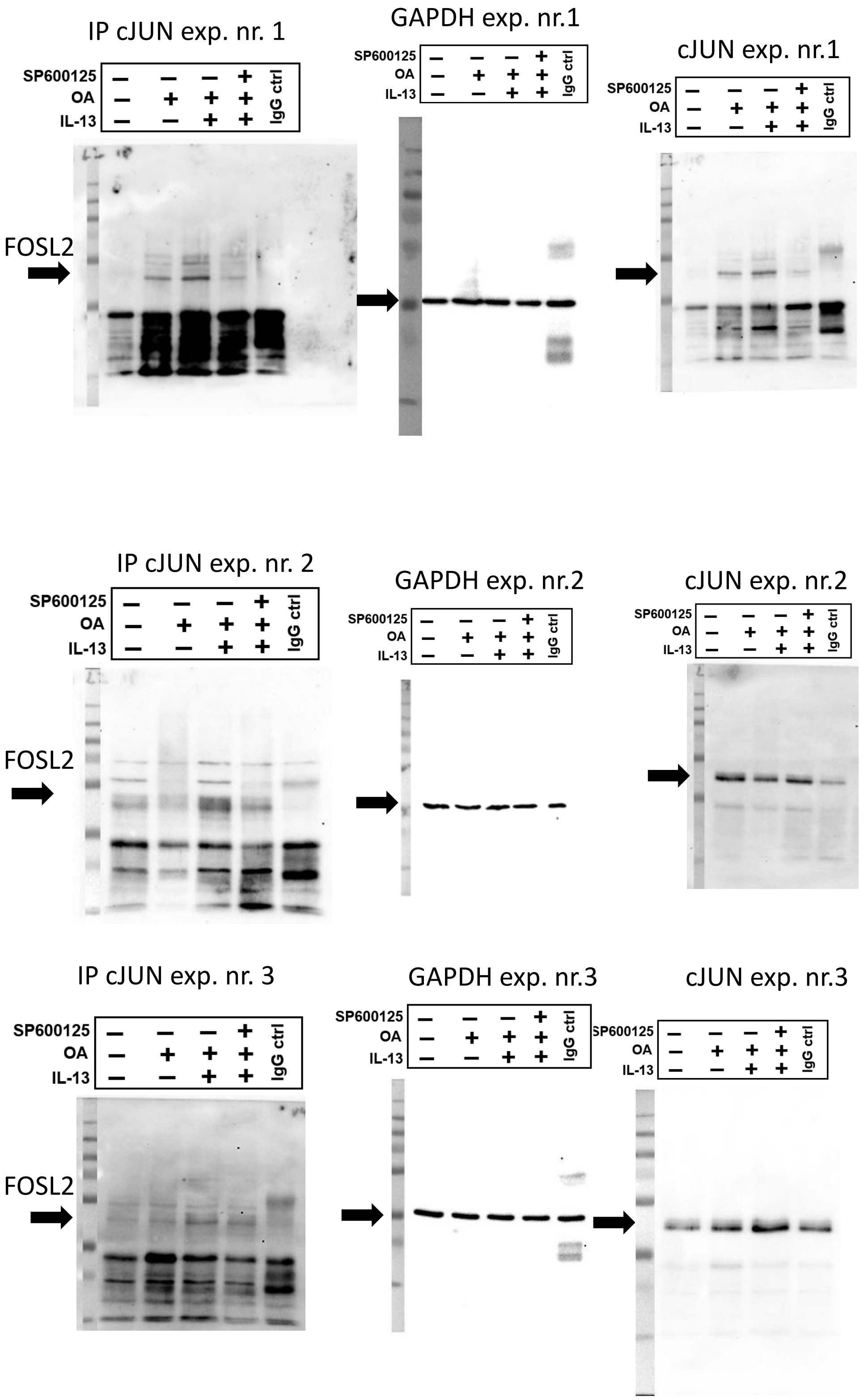
IP cJUN exp. nr. 1
GAPDH exp. nr.1
cJUN exp. nr.1
IP cJUN exp. nr. 2
GAPDH exp. nr.2
cJUN exp. nr.2
IP cJUN exp. nr. 3
GAPDH exp. nr.3
cJUN exp. nr.3
SP600125 – – – +
OA – + + +
IL-13 – – + +
IgG ctrl
FOSL2

Figure 5 J

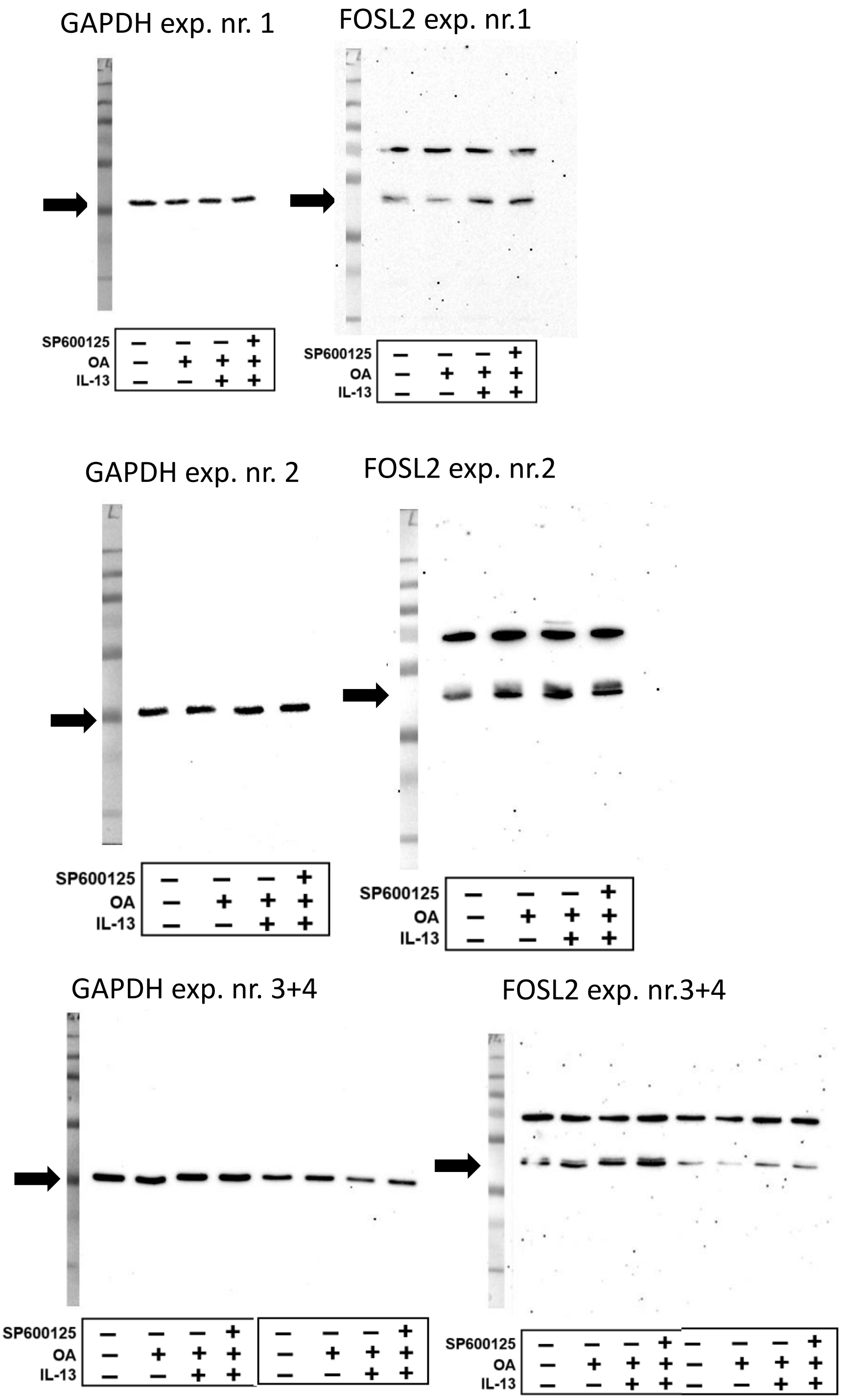

GAPDH exp. nr. 1
FOSL2 exp. nr.1
SP600125
OA
IL-13
SP600125
OA
IL-13
GAPDH exp. nr. 2
FOSL2 exp. nr.2
SP600125
OA
IL-13
SP600125
OA
IL-13
GAPDH exp. nr. 3+4
FOSL2 exp. nr.3+4
SP600125
OA
IL-13
SP600125
OA
IL-13

Figure 6 I

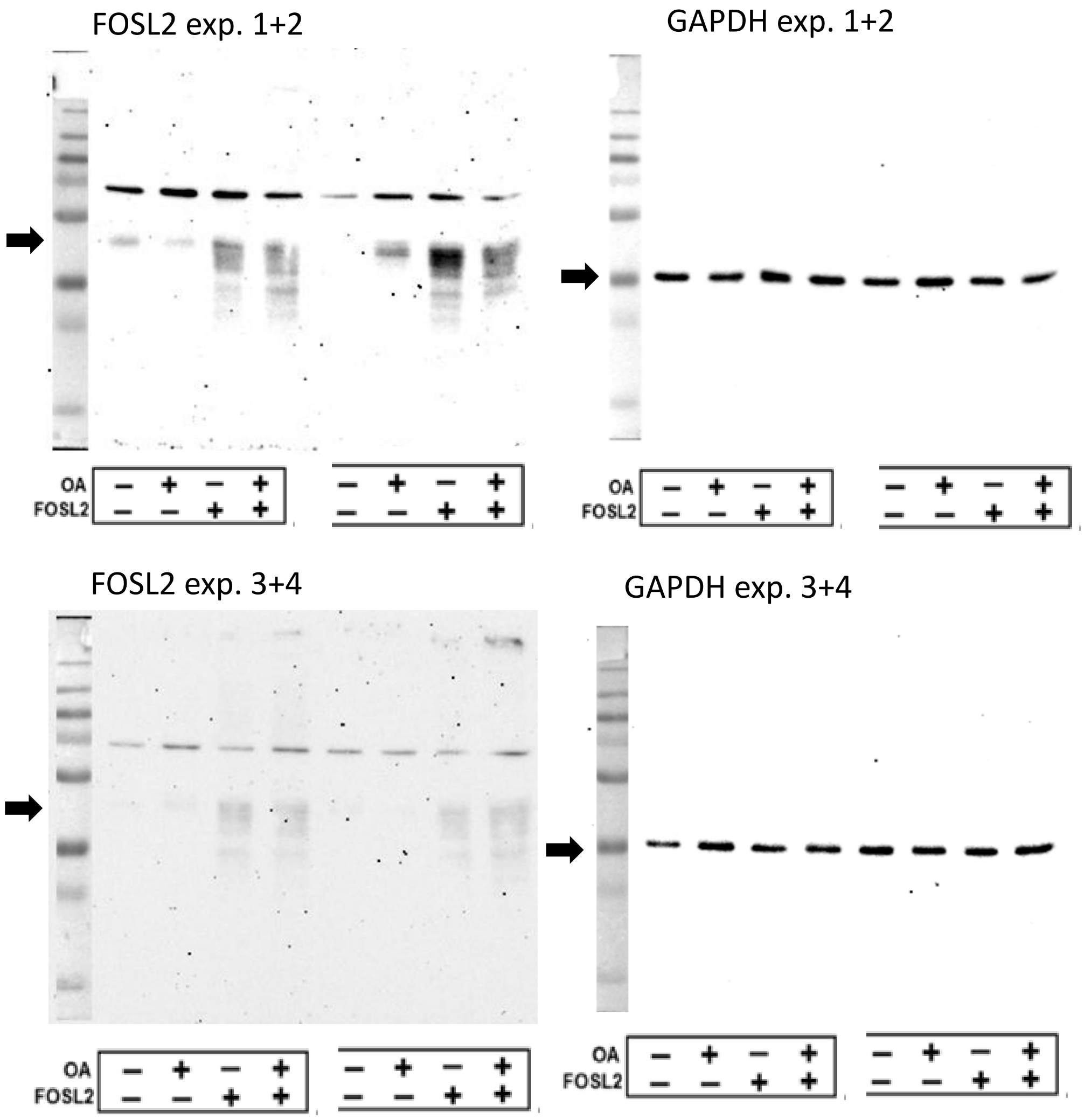
FOSL2 exp. 1+2
GAPDH exp. 1+2
OA
FOSL2
FOSL2 exp. 3+4
GAPDH exp. 3+4
OA
FOSL2

Figure 7 C

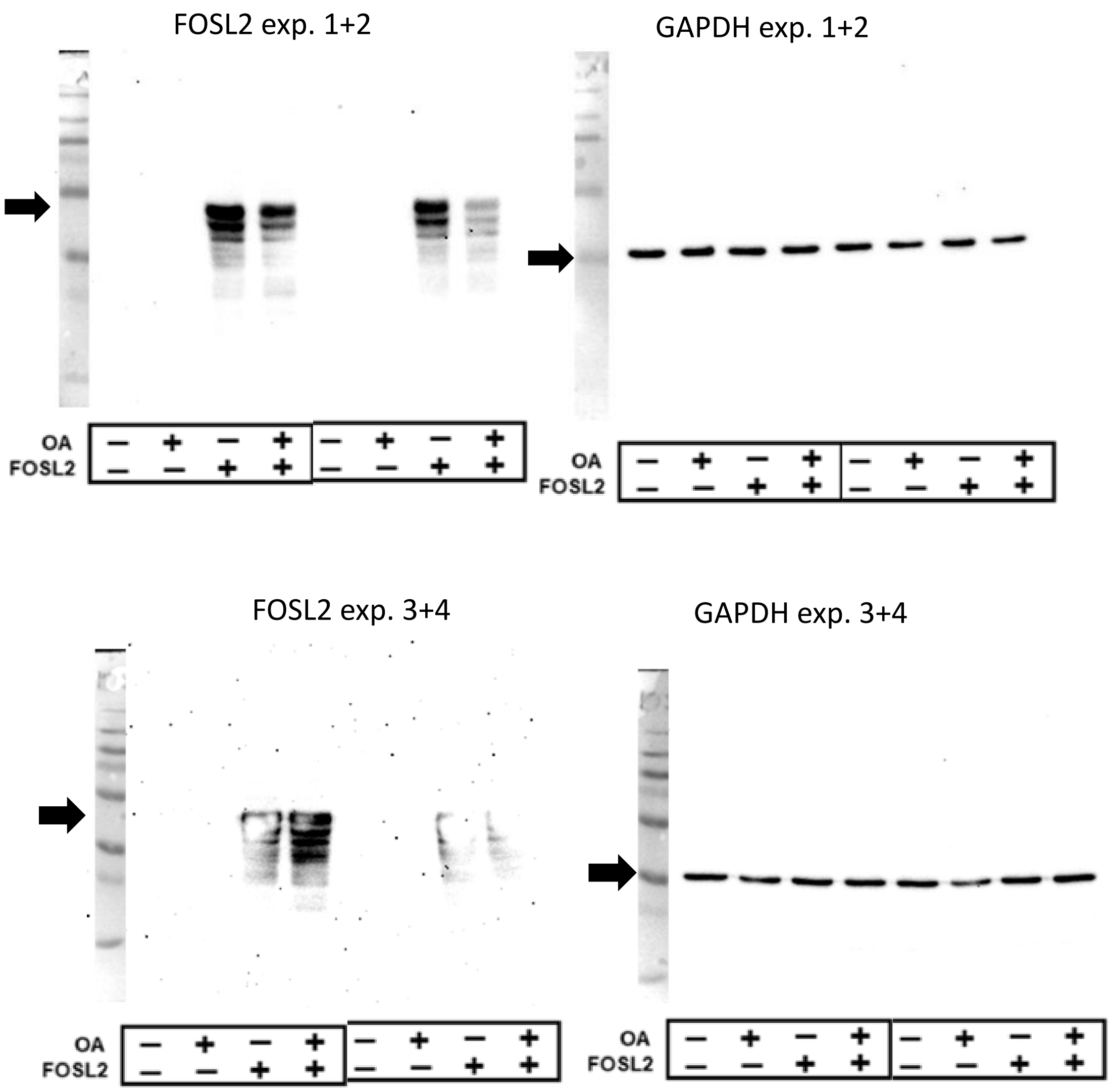
FOSL2 exp. 1+2
GAPDH exp. 1+2
OA
FOSL2
FOSL2 exp. 3+4
GAPDH exp. 3+4
OA
FOSL2
